# One-Pot Biosynthesis of Acetone from Waste Poly(hydroxybutyrate)

**DOI:** 10.1021/acssuschemeng.4c00357

**Published:** 2024-05-05

**Authors:** Benjamín O. Armijo-Galdames, Joanna C. Sadler

**Affiliations:** Institute of Quantitative Biology, Biochemistry and Biotechnology, School of Biological Sciences, University of Edinburgh, Roger Land Building, Alexander Crum Brown Road, King’s Buildings, Edinburgh EH9 3FF, U.K.

**Keywords:** bioplastics, biodegradable plastics, acetone, plastic upcycling, poly(hydroxyalkanoates), microbial biotechnology, Escherichia coli

## Abstract

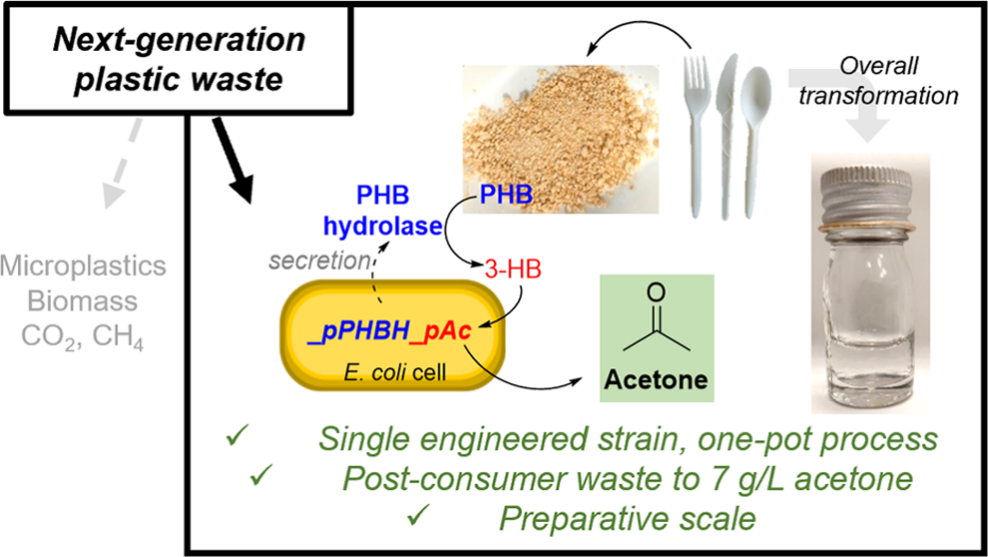

The plastic waste crisis is catalyzing change across the plastics life cycle. Central to this is increased production and application of bioplastics and biodegradable plastics. In particular, poly(hydroxybutyrate) (PHB) is a biodegradable bioplastic that can be produced from various renewable and waste feedstocks and is a promising alternative to some petrochemical-derived and non-biodegradable plastics. Despite its advantages, PHB biodegradation depends on environmental conditions, and the effects of degradation into microplastics, oligomers, and the 3-hydroxybutyrate (3-HB) monomer on soil microbiomes are unknown. We hypothesized that the ease of PHB biodegradation renders this next-generation plastic an ideal feedstock for microbial recycling into platform chemicals currently produced from fossil fuels. To demonstrate this, we report the one-pot degradation and recycling of PHB into acetone using a single strain of engineered *Escherichia coli*. Following strain development and initial bioprocess optimization, we report maximum titers of 123 mM acetone (7 g/L) from commercial PHB granules after 24 h fermentation at 30 °C. We further report biorecycling of an authentic sample of post-consumer PHB waste at a preparative scale. This is the first demonstration of biological recycling of PHB into a second-generation chemical, and it demonstrates next-generation plastic waste as a novel feedstock for the circular bioeconomy.

## Abstract 2

A bioprocess for converting next-generation plastic waste into a bulk chemical demonstrates a novel end-of-life option for biodegradable plastics that support the circular economy.

## Introduction

The plastic waste crisis has driven a rapid increase in the development and production of bioderived and biodegradable polymers,^1−4^ and the combined “bioplastics” production capacity is now projected to grow from 2.2 million tonnes per year in 2022 to approximately 6.3 million tonnes per year by 2027.^5^ With this, a sharp increase in the volume of post-consumer bioplastic waste is anticipated, prompting calls for mitigation of potential risks associated with its disposal through development of appropriate waste management technologies.^6−12^

One of the most well-known and widely used biodegradable bioplastics is poly(hydroxybutyrate) (PHB), a thermoplastic with potential applications in food packaging, materials, tissue engineering, and single-use foodware.^13^ PHB is a naturally occurring polyester comprising repeating units of 3-hydroxybutyrate (3-HB). It is synthesized by various microorganisms (e.g., *Cupriavidus necator*, *Alcaligenes latus*, *Methylocystis parvusstr. OBBP, Halomonas* spp., and *Bacillus megaterium*)^14−16^ from acetyl-CoA via three enzyme-catalyzed steps and serves as a carbon storage mechanism under nutrient-limited conditions. Intracellular PHB can then be utilized by the host under stress conditions, for example, by providing a carbon source under starvation conditions. Interestingly, PHB degradation products 3-HB and its oligomers have also been shown to exert a protective effect against protein aggregation and cellular damage from oxidative and heat stress.^17^ PHB can also be synthesized by non-native hosts via heterologous expression of the *phaCAB* operon^18^ and its mechanical properties controlled by choice of chassis, production medium, carbon source, fermentation mixing and aeration and integration into blends,^19^ and copolymers.^20,21^

**Figure 1:**
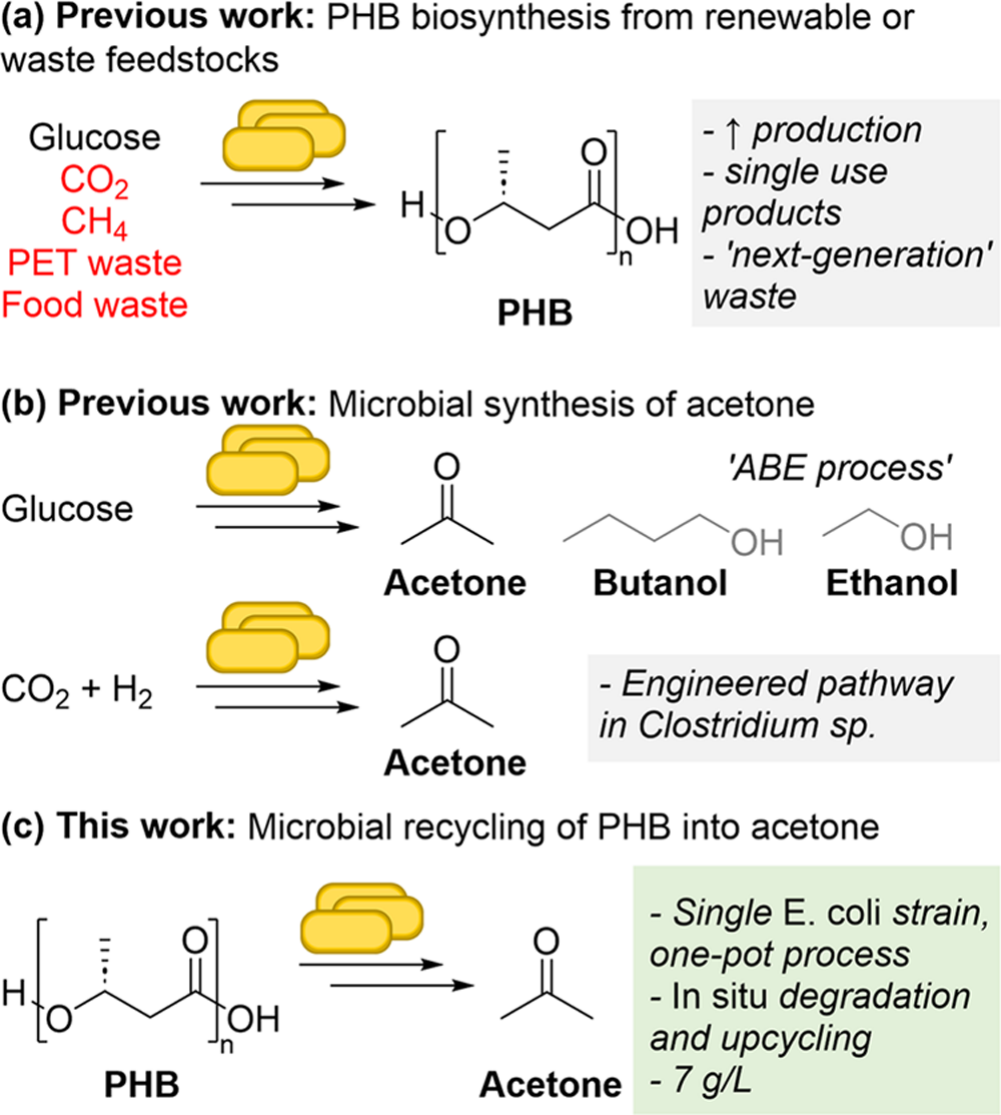
(a) PHB has been synthesized by engineered microorganisms using renewable (e.g., glucose) and waste (shown in red) feedstocks as the sole carbon source. (b) Acetone has been produced via microbial fermentation. (c) This work: one-pot degradation and upcycling of PHB into acetone with a maximum titer of 7 g/L acetone.

The ability of microorganisms to be cultivated using a diverse array of carbon sources and recent interest in waste valorization has inspired the development of a multitude of biobased systems to upcycle waste streams such as poly(ethylene terephthalate),^22−24^ agricultural waste,^25^ carbon dioxide,^26−29^ methane,^30^ and coffee grounds^31^ into PHB (Figure 1a).^32^ However, to date, little consideration has been given to the fate of PHB after consumer use. PHB degrades in certain natural environments via microbial metabolism,^33,34^ with approximately 20% of PHB-derived carbon converted into biomass and the remaining 80% released as carbon dioxide (aerobic conditions) or methane (anaerobic conditions).^35^ Inspired by recent studies that upcycle post-consumer petrochemical plastic waste^36−42^ and motivated to develop technologies to ensure a sustainable future for next-generation plastics, we hypothesized that the propensity of PHB for biodegradation makes it an ideal feedstock for rationally designed biodegradation and recycling systems to produce useful chemicals.

To demonstrate this, we herein report the engineering of a single strain of *Escherichia coli*, which simultaneously degrades PHB and recycles the resulting monomer 3-HB into the solvent acetone. Acetone is used extensively across the chemical, cosmetic, and paint industries. It had a market value in excess of $6bn in 2022, with the production volumes exceeding 7 million tonnes per year using oil-derived feedstocks. Current production technologies are hugely energy-intensive, predominantly relying on propene cracking or reforming processes, which together produce 2.55 kg CO_2_e/kg acetone.^43^ Development of sustainable acetone production technologies is therefore a research priority in enabling a sustainable chemical industry. Biotechnological approaches using renewable feedstocks such as glucose are a promising alternative that alleviate reliance on fossil fuels and have been demonstrated at both laboratory and industrial scales (Figure 1b). For example, using glucose-fed batch cultures of engineered *E. coli*, acetone titers of up to 122 mM have been reported at a laboratory scale.^44−46^ Acetone can also be coproduced with ethanol and butanol via the acetone–butanol–ethanol (ABE) fermentation process, which is currently performed industrially for biofuel production.^47^ A continuous process for acetone (2.5 g/L/h) and isopropanol (3 g/L/h) syntheses from carbon dioxide and hydrogen has also been developed, demonstrating the potential of biotechnology for more sustainable acetone production at an industrial scale.^43^ Inspired by these studies, we set out to develop a complementary bioprocess to produce acetone from PHB (Figure 1c) that would demonstrate the potential of next-generation plastics as a feedstock for the circular bioeconomy.

## Results and Discussion

**Figure 2:**
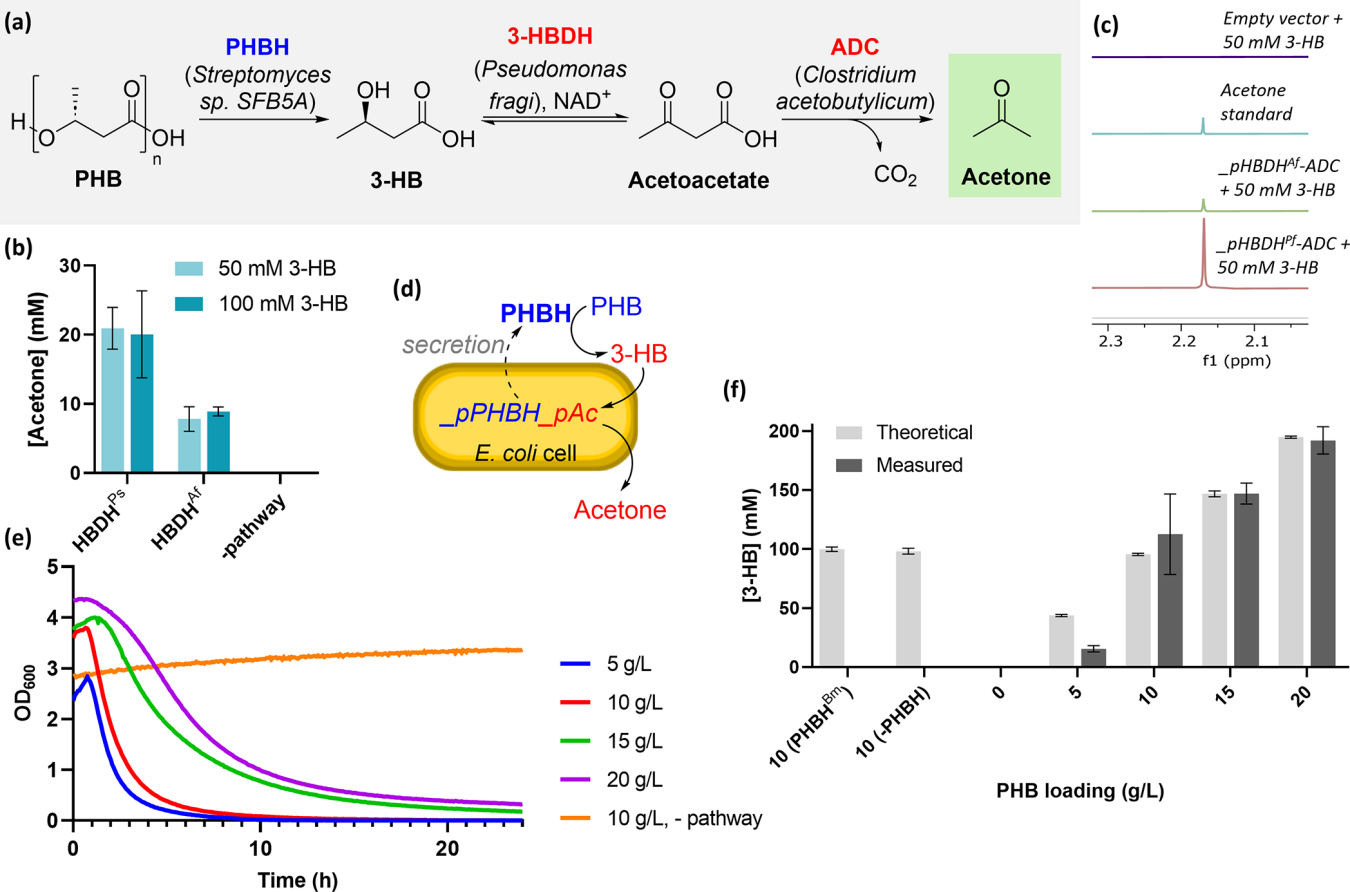
(a) PHB to acetone pathway in engineered *E. coli*. (b) Acetone production from 3-HB using *E. coli*_pHBDH^Af/Pf^-ADC via whole cell biotransformation. Conditions: cells resuspended in M9-glucose supplemented with 3-HB, OD_600_ = 8, 22 °C, 24 h. (c)^1^H NMR spectra of chloroform-extracted cultures from fermentation experiments with *E. coli*_pRSF-Duet1 (empty vector) or *E. coli*_pHBDH^Af/Pf^ in comparison to an acetone standard. Full spectra are shown in Figure S1. (d) Coexpression of pPHBH and pAc for *in situ* PHB degradation (blue) and conversion to acetone (red). (e) Turbidimetric PHB degradation assay by supernatant of *E. coli*_pPHBH_pAc. Conditions: filter-sterilized supernatant from the expression culture added to PHB, then incubation at 30 °C, 60 h. (f) 3-HB quantification of turbidimetric degradation assay samples after 60 h incubation showing theoretical 3-HB yield corresponding to 100% degradation of PHB (light gray) and measured 3-HB (dark gray). Conditions: filter-sterilized supernatant from the expression culture added to PHB, then incubation at 30 °C, 60 h.

Our initial objective was to construct a strain of *E. coli* capable of converting PHB depolymerization product 3-HB into the target molecule acetone. For this proof-of-concept study, we chose *E. coli* BL21 (DE3) as the chassis, which is widely used for heterologous protein expression, whole cell biocatalysis, and fermentations. We envisaged that 3-HB could be converted to intermediate acetoacetate by an NAD^+^-dependent 3-hydroxybutyrate dehydrogenase (3-HBDH), followed by decarboxylation catalyzed by acetoacetate decarboxylase (ADC) to generate acetone (Figure 2a). Three 3-HBDH candidate enzymes were screened in combination with ADC from *Clostridium acetobutylicum* (ADC^Ca^)^44,46^ for protein expression and pathway activity. These were 3-HBDH from *Alcaligenes faecalis* (3-HBDH^Af^),^48^*Pseudomonas fragi* (3-HBDH^Pf^),^49^ and *Ralstonia pickettii* (3-HBDH^Rp^),^50^ which are all reported to express well in *E. coli* and have activity at mesophilic temperatures. While 3-HBDH^Rp^ was poorly expressed and did not produce acetone, 3-HBDH^Af^ and 3-HBDH^Pf^ were both expressed in soluble form, and when coexpressed with ADC^Ca^, they produced 8 ± 2 and 21 ± 3 mM acetone from 50 mM 3-HB, respectively (Figure 2b); acetone production was also confirmed by NMR spectroscopy (Figures 2c and S1). It is noteworthy that isopropyl alcohol was not detected (Figure S2), indicating that conversion to the corresponding alcohol *via* endogenous *E. coli* reductases was not decreasing acetone titers. Interestingly, analysis of protein expression by SDS-PAGE showed 3-HBDH^Pf^ to have much lower expression levels than 3-HBDH^Af^ (Figure S3) yet resulted in 2.7-fold higher acetone titers.

Encouraged by these data, we next investigated whether the 3-HB to acetone pathway could be integrated with *in situ* PHB degradation (Figure 2d). We hypothesized that a PHB hydrolase (PHBH) could be secreted into the reaction medium *via* genetic fusion of an enhanced PelB (PelB1) secretion tag,^51^ enabling extracellular depolymerization of PHB into 3-HB, which in turn would serve as a substrate for intracellular 3-HBDH. PHBH candidates from *B. megaterium* (PHBH^Bm^) and *Streptomyces* sp. *SFB5A* (PHBH^St^) were selected for screening. PHBH^Bm^ is reported to degrade a broad range of PHB granule types, including natively bioproduced granules coated in membrane proteins, as well as amorphous, denatured granules.^52^ PHBH^St^ was isolated from decayed hardwood mulch and was selected for its ability to degrade PHB into 3-HB monomers as the major degradation product at 37 °C and neutral pH, whereas many other known PHBHs produced a mixture of monomers and oligomers.^53^ The candidate genes together with an N-terminal PelB1 secretion tag^51^ were cloned into an isopropyl β-d-1-thiogalactopyranoside (IPTG)-inducible expression vector (generating plasmid pPHBH^St/Bm^) and expressed in *E. coli* BL21 (DE3). Extracellular expression of PHBH^St^ was detected by SDS-PAGE, whereas PHBH^Bm^ was expressed only intracellularly (Figure S4). This was confirmed by turbidimetric analysis of the cell-free expression medium to which powdered PHB was added. PHB degradation was clearly observed for PHBH^St^ but not for the negative controls and PHBH^Bm^ (Figure S5). Encouraged by these data, we cotransformed *E. coli* BL21 (DE3) with the plasmid encoding ADC^Ca^ and 3-HBDH^Pf^ (hereafter referred to as pAc) and pPHBH^St^ to generate the full pathway strain *E. coli*_pPHBH_pAc. Protein expression was induced for 24 h before collecting the supernatant for use in PHB degradation assays. Gratifyingly, 97% PHB degradation of up to 10 g/L PHB was achieved in the first 8 h, while PHB loading of 15 and 20 g/L achieved a 71 and 67%, respectively (Figures 2e and S6). Furthermore, derivatization of degradation products with 3-nitrophenylhydrazine confirmed quantitative conversion to the 3-HB monomer under these conditions (Figure 2f).

With a PHB depolymerization method in hand, we next investigated whether this could be integrated with the acetone production pathway. The addition of induced *E. coli_*pAc cells to the supernatant of PHB degradation cultures provided 11 ± 1 mM acetone (23% conversion), which was comparable to 13 ± 3 mM (26% conversion) acetone produced from the addition of 50 mM 3-HB to induced *E. coli_*pAc cells (Figure S7).

**Figure 3:**
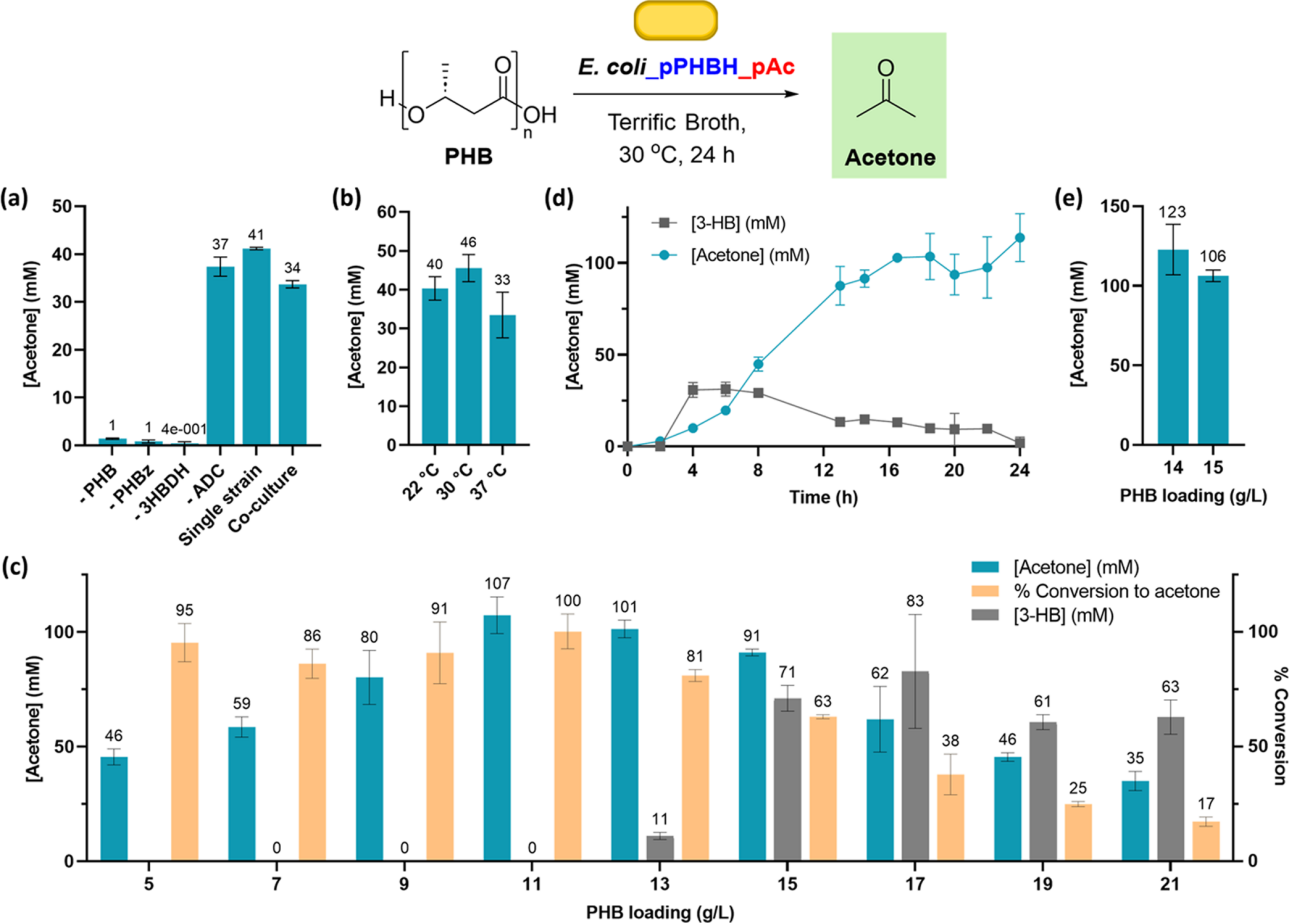
(a) Acetone production from PHB *via* a single strain or a co-culture method. Conditions: 5 g/L PHB, TB media, 37 °C pre-induction temperature, followed by addition of 0.1 mM IPTG and 0.2% v/v arabinose, and post-induction incubation at 37 °C for 24 h. For co-cultures, 2.5 mL of each single strain culture following induction was added directly to PHB and incubated at 37 °C for 24 h. Control experiments lacking PHBH, 3-HBDH, or ADC had PHB loadings of 6 g/L. (b) Temperature screening for one-pot PHB upcycling. Conditions: 5 g/L PHB, TB media, 37 °C pre-induction temperature, followed by addition of 0.1 mM IPTG and 0.2% v/v arabinose, and post-induction incubation for 24 h. (c) Acetone titers from the PHB loading experiment. Conditions: TB media, 37 °C pre-induction temperature, followed by addition of 0.1 mM IPTG and 0.2% v/v arabinose, and post-induction incubation at 30 °C for 24 h. (d) Time course of acetone production and 3-HB quantification under optimized conditions. Conditions: 11 g/L PHB, 37 °C pre-induction temperature, followed by addition of 0.1 mM IPTG and 0.2% v/v arabinose, and post-induction incubation at 30 °C for 24 h. (e) Acetone production from PHB after 48 h incubation. Conditions: TB media, 37 °C pre-induction temperature, followed by addition of 0.1 mM IPTG and 0.2% v/v arabinose, and post-induction incubation at 30 °C for 48 h.

We next investigated whether PHB depolymerization and 3-HB upcycling could be carried out in a one-pot process. We compared the use of a strain of *E. coli* harboring both plasmids (*E. coli*_pHDBD_pAc) with a division of labor approach, comprising a co-culture of *E. coli*_pPHBH and *E. coli*_pAc, each also harboring an empty vector to ensure consistent antibiotic usage. Both systems were cultivated in the growth medium containing powdered PHB, and protein expression was induced at the mid-exponential phase. Prior to optimization, 41 ± 0.2 mM acetone was detected after 24 h, equating to 84% conversion from PHB (Figure 3a), outperforming the co-culture approach, which only produced 34 ± 1 mM acetone (69% conversion) (Figure 3a). All strains showed a similar PHB degradation efficiency (Figure S5), and we therefore discounted this as a cause for the lower acetone titers from the co-culture experiment. An alternative hypothesis is that different growth rates of the two strains in the co-culture lead to a less well-balanced ratio of PHBH to 3-HBDH and ADC,^54^ thereby decreasing the overall product titers. However, as a single strain process is preferable for future scale-up studies, this was not investigated further, and we proceeded to optimize the process with a single strain.

Control experiments showed no acetone production in the absence of PHB, PHBH, or HBDH. However, strains lacking ADC^Ca^ retained acetone production, albeit at a lower efficiency (Figure 3a, 37 ± 2 mM, 64% conversion from PHB). Intrigued by this result, we extracted the fermentation broth with CDCl_3_ for analysis by nuclear magnetic resonance (NMR) spectroscopy to determine whether acetoacetate decarboxylation was occurring under acetone assay conditions (Figure S8). This confirmed acetone to be present in the fermentation mixture, indicating either spontaneous, abiotic decarboxylation of acetoacetate during the fermentation or background biological reactivity such as an endogenous acetoacetate decarboxylase, which can increase the acetoacetate decarboxylation reaction rate by a factor of 5.3 × 10^9^ at 25 °C.^55^ Basic Local Alignment Search Tool (BLAST) analysis of the *E. coli* genome using ADC^Ca^ as the query sequence identified a putative acetoacetate decarboxylase, which could be involved in acetone formation. Regardless, as conversions were 1.3-fold higher with overexpression of the heterologous decarboxylase, we decided to proceed with process optimization with ADC^Ca^ included in the full pathway strain.

Further optimization of fermentation conditions revealed Terrific Broth (TB) to yield the highest acetone titers from 3-HB compared to Lysogeny Broth (LB), minimal media, and 2YT (Figure S9). Increasing IPTG concentration, performing the process under reduced pressure, and addition of glycerol, which has been reported to decrease cellular stress while providing additional carbon for metabolism,^51,56^ did not lead to further improvements (Figures S10–S12). However, fermentation temperature did have a positive effect on acetone titers, with a maximum titer of 46 ± 3 mM (95% conversion from PHB) being obtained after 24 h upon decreasing the fermentation temperature from 37 to 30 °C (Figure 3b). Extracellular protein expression with the enhanced PelB1 tag has been reported to be highest at 20 °C,^51^ while the pathway enzymes have optimum activity between 30 and 45 °C. The observed improvement in acetone titer at 30 °C was therefore theorized to be a balance between protein expression and biocatalytic activity.

With these improved assay conditions in hand, we sought to determine the maximum concentration of acetone that could be achieved using this system. To this end, *E. coli*_pPHBH_pAc was cultivated with PHB powder loadings ranging from 5 to 21 g/L. A maximum acetone titer of 107 ± 8 mM (100% conversion from 11 g/L PHB) was obtained after 24 h, with acetone titers decreasing with PHB loadings of 13 g/L and above (Figure 3c). To explore this, we performed growth challenge assays of *E. coli*_pPHBH_pAc exposed to 0–200 mM acetone or 3-HB (Figures S13 and S14), which showed that 3-HB was more toxic to cells than acetone, with a 24% decrease in the average growth rate at 40 mM loading and final OD_600_ values >1 for concentrations exceeding 140 mM. Conversely, acetone was surprisingly well tolerated, with a significant (*p* = 0.0117) decrease in the growth rate only observed at concentrations of 120 mM and above (Figures S13 and S14) and OD_6oo_ > 1 after 24 h for all acetone concentrations. This observation is in line with a previous study reporting the growth of *E. coli* DH5α and MG1655 to OD_600_ > 2 in 300 mM acetone, which in combination with our data suggests that acetone toxicity is not a major bottleneck to the pathway.^57^ We also explored the alternative hypothesis that acetoacetate was being consumed as a carbon source for microbial growth, thereby accounting for loss of yield at higher PHB loadings.^58^ To test this, we measured the growth of strains expressing no pathway enzymes (_pRSF), the full pathway (_pAc), or the 3-HB dehydrogenase only (_pHBDH^Pf^) in minimal media containing 3-HB as the sole carbon source, theorizing that growth would be indicative of diversion of 3-HB-derived carbon flux toward biomass accumulation. As shown in Figure S15, very slow growth was observed for _pHBDH^Pf^, but no significant growth of the full pathway strain _pAc was detected. This is suggestive of biomass accumulation not being a major draw on 3-HB-derived C flux through the pathway.

Drawing these data together, we hypothesized that rapid depolymerization of PHB (Figure 2e) led to 3-HB concentrations high enough to exert significant growth rate inhibition, thereby decreasing pathway enzyme production and ultimately leading to the observed lower acetone titers as PHB loading was increased above 13 g/L. As such, fine-tuning of relative expression levels of PHBH, 3-HBDH, and ADC will be a focus of future work.

We further characterized the process *via* a time course experiment. This showed full conversion to acetone after 24 h, concurrent with full consumption of 3-HB (Figure 3d). Increasing the incubation time to 48 h for higher PHB loadings (Figure 3e) increased the acetone titer to 123 ± 16 mM (92% conversion from 14 g/L PHB loading) and 106 ± 4 mM (74% conversion from 15 g/L PHB loading), representing 1.2-fold increase in acetone titers at these higher PHB loadings. Increasing the reaction time further to 96 h and overexpression of an NAD^+^ oxidase enzyme to increase intracellular NADH levels^59^ showed no further improvement (data not shown).

**Figure 4:**
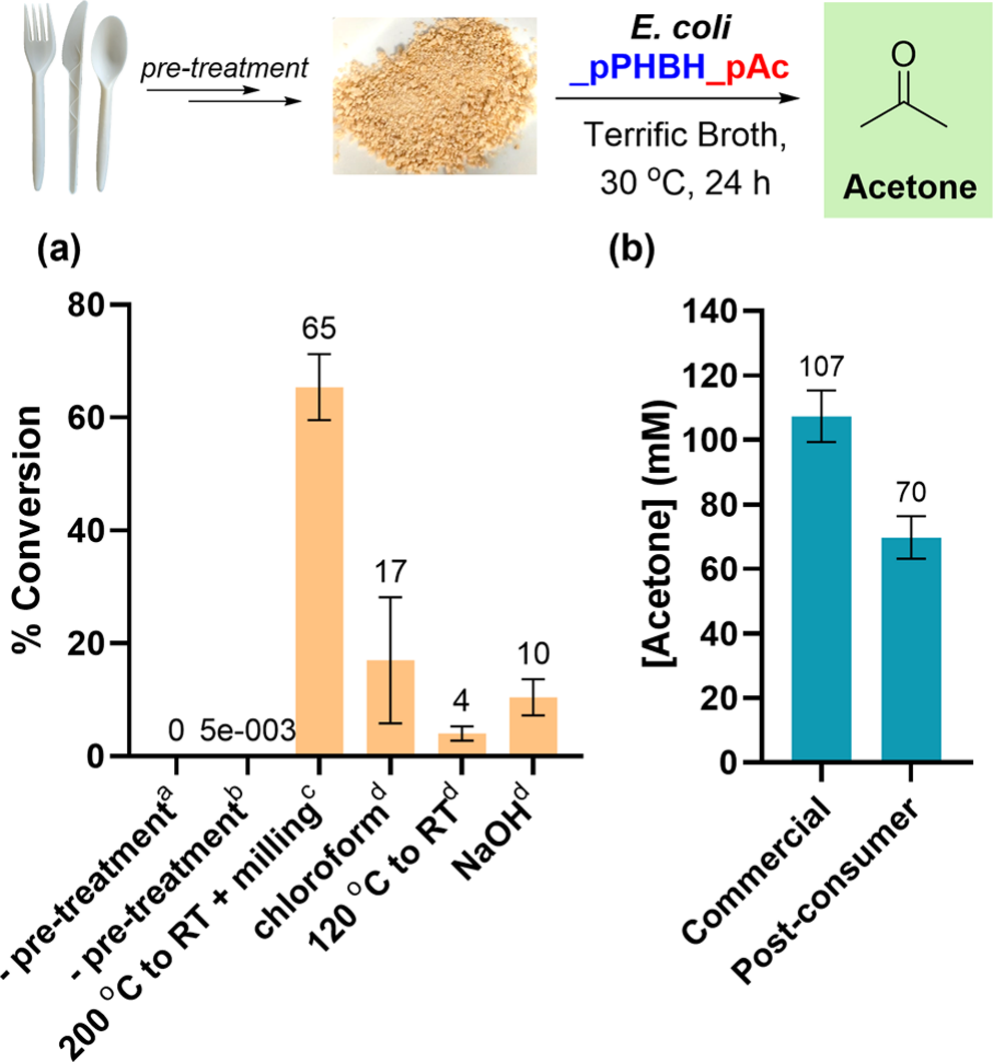
(a) Screen of the effect of post-consumer PHB pre-treatment conditions on acetone production. Conditions: ^a^6 g/L PHB, 30 °C, 24 h; ^b^6 g/L PHB, 30 °C, 48 h; ^c^11 g/L PHB, 30 °C, 24 h; and ^d^5 g/L PHB, 30 °C, 168 h. (b) Comparison of acetone production using commercial and post-consumer PHB at an analytical scale. Conditions: 11 g/L PHB, 30 °C, 24 h.

Having developed a strain for simultaneous PHB degradation and conversion into acetone, we next set out to demonstrate its utility on an authentic post-consumer PHB waste cutlery sample. A range of pre-treatment processes were tested,^60−62^ with melting, cooling, and then milling into a fine powder being the most effective for subsequent conversion into acetone (Figure 4a). At a 5 mL scale, we achieved 70 ± 7 mM acetone (65% conversion) at a PHB loading of 11 g/L of cutlery waste compared to 100% conversion for commercial PHB feedstock (Figure 4b). A high degree of residual PHB was observed for other pre-treatments in which lower titers of acetone were obtained, leading us to hypothesize that these methods did not decrease the degree of PHB crystallinity sufficiently to enable efficient depolymerization by PHBD.^63,64^

**Figure 5:**
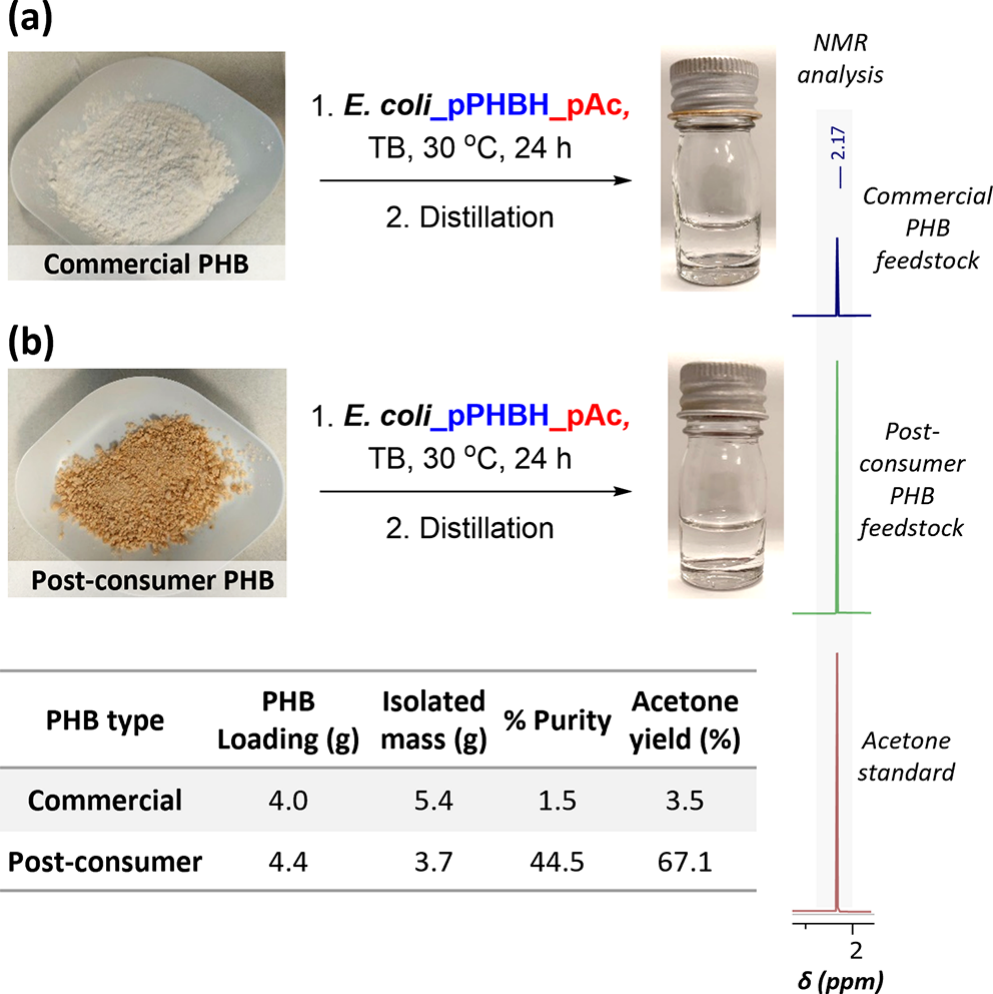
Preparative scale recycling of PHB into acetone using (a) commercial PHB powder and (b) pre-treated post-consumer PHB cutlery. Conditions: 400 mL culture volume, Terrific Broth (TB) media, 11 g/L PHB loading, 30 °C post-induction temperature for 24 h, and then product recovery by rotary evaporation. Full NMR spectra are available in the Supporting Information (Figure S16).

Encouraged by these data, we scaled up the process to a preparative scale, with the goal of recovering acetone from cultures by distillation. Commercial or pre-treated post-consumer PHB waste was added to 400 mL cultures of *E. coli*_pPHBH_pAC following pathway induction with IPTG and incubated at 30 °C for 24 h. Analysis of the supernatant using the colorimetric assay showed 107 ± 2 and 78 ± 7 mM acetone from the commercial and post-consumer samples, respectively (data not shown), prompting us to attempt product recovery by rotary evaporation (Figure 5). Pleasingly, analysis of the distillate by NMR spectroscopy showed acetone as a major component (Figures 5 and S16). Analysis of acetone content by reference to an internal standard showed 3.5 and 67.1% recovery of acetone from commercial and post-consumer PHB feedstocks, respectively. These data demonstrate the exciting potential for a novel, scalable process to generate acetone from next-generation waste, with improvements to product purities expected upon fine-tuning of distillation methods upon further scale-up.

## Conclusions

Use of biodegradable plastics as a feedstock for the circular bioeconomy has been overlooked in favor of petrochemical-derived polymeric materials. However, given the projected increase in production and use of bioplastics, it is necessary to consider end-of-life upcycling opportunities at the outset to ensure that next-generation plastic products are sustainable by design and not contributing to “pollution swapping”.^7^ To this end, this work describes the first microbial process for recycling the common bioplastic PHB into a single chemical product. We report a single strain of *E. coli* capable of simultaneously depolymerizing up to 11 g/L commercial and post-consumer PHB into its constituent monomer, 3-HB, and converting it into the bulk chemical and solvent acetone with maximum titers of 123 ± 16 mM (92% conversion from 14 g/L loading PHB, Figure 3e), demonstrating the potential for next-generation plastics to serve as a feedstock for the emerging circular bioeconomy. We demonstrate this process at a preparative scale, recovering 1.6 g of acetone from 4.4 g of post-consumer PHB waste before any process optimization, and anticipate that further improvements including *in situ* product recovery could be achieved upon scale-up. While full life cycle analysis (LCA) is premature at the present stage of technology development, the most significant cost factors of this bioprocess are predicted to be PHB pre-treatment, media costs, and acetone recovery.^65^ Development of scalable strategies to address this, such as alternative pre-treatment methods and use of carbon-rich waste streams as microbial growth media, will form the basis of future work prior to scale-up and detailed LCA studies.

## Materials and Methods

### Construction of pPHBH and pAc

All genes used in this study (Table S1) were ordered as lyophilized double-stranded DNA (Thermo Fisher or Twist Biosciences) and resuspended to 10 ng/μL in Milli-Q-H_2_O prior to use. 3-HBDH and ADC were cloned into pRSF-Duet1 using NcoI & *Hin*dIII and NdeI & XhoI restriction sites, respectively. For PHBH^St/Bm^, genes were cloned into pET22b using NdeI and XhoI restriction sites. 5 μL of each ligation reaction was used to transform *E. coli* DH5α *via* the heat-shock method before plating out onto LB-agar plates containing kanamycin (50 μg/mL) for pRSF-Duet1 transformants or ampicillin (100 μg/mL) for pET22b transformants. Plasmid DNA was amplified by MiniPrep and the sequence verified by Sanger sequencing. *E. coli*_pPHBH_pAc was generated by cotransformation of BL21 (DE3) with pPHBH^Bm^ and pRSF-Duet1-HBDH^Pf^-ADC (pAc) *via* the heat-shock method using 100 ng of each plasmid and plated out onto LB-agar selection plates containing kanamycin (50 μg/mL) and ampicillin (100 μg/mL). All plasmids and strains described in this study are detailed in Tables S2 and S3.

### One-Pot PHB Degradation and Upcycling

LB containing kanamycin (50 μg/mL) and ampicillin (100 μg/mL) was inoculated with a single colony from a freshly streaked plate of *E. coli*_pPHBH_pAc and incubated overnight at 37 °C with orbital shaking. The next day, Terrific Broth (TB) was inoculated to an initial OD_600_ of 0.05 and incubated at 37 °C and 200 rpm (2.5 cm orbital throw). When the culture reached an OD_600_ of 0.5–0.6, protein expression was induced with IPTG (0.1 mM final concentration), and arabinose (0.2% w/v final concentration) was added. For analytical scale experiments, 5 mL of the induced culture was transferred to a 50 mL Falcon tube containing 5–21 g/L PHB (Sigma-Aldrich, CAS 29435–48–1) and cultures were incubated at 30 °C, 200 rpm (2.5 cm orbital throw) for 24 h. For preparative scale experiments, PHB (11 g/L) was added to a 400 mL induced culture, which was then incubated at 30 °C, 200 rpm (2.5 cm orbital throw) for 24 h. Cultures were then clarified by centrifugation (10 min, 3200*g*, 4 °C) and the supernatant analyzed for 3-HB and acetone concentration (analytical scale) or, for preparative scale experiments, the acetone recovered by rotary evaporation (2 h, 65 mbar, 280 rpm, 40 °C water bath).

### Acetone Quantification Assay

Colorimetric detection of acetone was performed based on the protocol described by Nielsen et al.^66^ Briefly, 125 μL of the culture supernatant was added to an Eppendorf tube, then vanillin (75 μL of a 100 mM stock in 25% ethanol) and NaOH (50 μL of an aq 5 M NaOH stock) were added, and samples were immediately incubated at 60 °C for 10 min and then cooled to 22 °C for 10 min. 200 μL of the reaction mixture was transferred to a 96-well plate, and the absorbance at 430 nm was measured (SPECTROstar Nano Microplate Reader, BMG Labtech). A sample of the culture supernatant incubated under the same conditions without a PHB substrate was included in each experiment for background subtraction. Finally, acetone concentration was calculated by reference to a calibration curve prepared using 0–50 mM acetone standards (Figure S17).

### PHB Degradation Assay

Strains were cultured in 10 mL of LB containing kanamycin (50 μg/mL) and ampicillin (100 μg/mL) as described above. After 24 h of post-induction incubation, cells were centrifuged (10 min, 3200*g*, 4 °C) and the extracellular fraction was collected and filter-sterilized. Streptomycin (50 μg/mL) was added to prevent further cell growth. 600 μL of the resulting supernatant was transferred to a 24-well plate containing PHB (5 to 20 g/L). A control experiment without PHB was included for background subtraction. The reaction was incubated at 30 °C, 300 rpm orbital shaking in a Plate Reader (SPECTROstar Nano Microplate Reader, BMG Labtech), measuring absorbance at 600 nm every 5 min for 60 h.

### 3-HB Quantification Assay

3-HB was derivatized with 3-nitrophenylhydrazine (NPH) prior to analysis by high-performance liquid chromatography (HPLC).^67,68^ Briefly, a 150 μL fermentation sample was quenched with 150 μL of acetonitrile containing 0.2% v/v trifluoroacetic acid (TFA), vortexed, and incubated for 30 min at 4 °C. Samples were clarified by centrifugation (10 min, 15,000*g*, 4 °C) and the supernatant concentrated by evaporation. 50 μL of this mixture was transferred to a fresh Eppendorf tube and mixed with 1-ethyl-3-(3-(dimethylamino)propyl) carbodiimide (50 μL from a 150 mM stock in methanol) and pyridine (50 μL from a 6% v/v stock in 75% methanol). The reaction was incubated at 40 °C for 15 min before adding NPH (50 μL from a 200 mM stock in 75% methanol) and incubated again at 40 °C for 60 min. Then, 570 μL of Milli-Q-H_2_O was added and the derivatized samples were stored at 4 °C until analysis by HPLC (see the Supporting Information Section 1.5).

### Preparation of Post-consumer PHB

Post-consumer PHB waste (AirCarbon, Newlight Technologies) was heated to 200 °C for 30 min before cooling to 22 °C and breaking into fragments, which were stored at −80 °C for 20 min and then immediately ground into a fine powder using a pestle and mortar. The powder was then used without further modification for the acetone production experiments.

## Supporting Information Available

General Materials and Methods section contains molecular biology techniques; protocols for toxicity assays and use of 3-HB as a sole carbon source; NMR spectroscopy analysis and HPLC sample preparation from fermentation samples; the additional experimental data contain NMR spectra for acetone production and isopropanol reduction; SDS-PAGE of 3-HBDH and PHBH expressions; PHB degradation assay; optimization of fermentation process (fermentation media, effect of IPTG, reduced pressure, additives supplementation); growth challenge assays with 3-HB and acetone; 3-HB as a sole carbon source growth curves; calibration curve for colorimetric assay; 3-HB quantification *via* HPLC and exemplar chromatograms; List of Sequences, Plasmids, and Strains section contains a summary of the sequences, plasmids, and strains (PDF)
