# Supplementary material for: One-Pot Biosynthesis of Acetone from Waste Poly(hydroxybutyrate)
Source: ACS Sustain Chem Eng. 2024 May 5;12(20):7748–56. doi: 10.1021/acssuschemeng.4c00357 (PMC11110063; doi:10.1021/acssuschemeng.4c00357)
Supplement: Supplementary file 1 — sc4c00357_si_001.pdf [file sc4c00357_si_001.pdf]

Supporting Information

One-pot biosynthesis of acetone from waste poly(hydroxybutyrate)

Benjamín O. Armijo-Galdames[1] and Joanna C. Sadler[1*]

[1]Institute of Quantitative Biology, Biochemistry and Biotechnology, School of Biological Sciences, University of Edinburgh, Roger Land Building, Alexander Crum Brown Road, King's Buildings, Edinburgh, EH9 3FF

*Corresponding author: joanna.sadler@ed.ac.uk

Number of pages: 22

Number of figures: 20

Number of tables: 3

Contents

1 General materials and methods 3
1.1 Molecular biology techniques 3
1.2 Toxicity assays 3
1.3 3-HB as a sole carbon source 4
1.4 Sample analysis via NMR spectroscopy 4
1.5 HPLC sample preparation from fermentation samples 4
2 Additional experimental data 5
2.1 NMR spectra for acetone production from 3-HB 5
2.2 Analysis of acetone reduction to isopropanol 6
2.3 SDS-PAGE analysis of 3-HBDH expression cultures 6
2.4 SDS-PAGE for PHB depolymerase expression 7
2.5 PHB degradation assay 7
2.6 PHB degradation time 8
2.7 Proof of concept of acetone production from PHB 8
2.8 NMR spectra of acetone production by *E. coli*_pHBDH 9
2.9 Fermentation media screening for acetone production from 3-HB 10
2.10 Additives screening for acetone production from 3-HB 10
2.11 Effect of IPTG concentration on acetone production from 3-HB 11
2.12 Effect of reduced pressure on acetone production from PHB 11
2.13 Growth challenge assays with 3-HB and acetone 12
2.14 Maximum derivative in toxicity assays with acetone and 3-HB 13
2.15 3-HB as sole carbon source growth curve 14
2.16 NMR spectra of distillate from preparative scale acetone production 15
2.17 Calibration curve for colorimetric detection of acetone 17
2.18 Calibration curve for 3-HB quantification 17
2.19 Exemplar HPLC chromatograms 18
3 List of sequences, plasmids and strains 19
3.1 Gene sequences 19
3.2 Plasmids 21
3.3 Strains 22
4 References 22

1 General materials and methods

Unless otherwise stated, starting reagents, nuclear magnetic resonance (NMR) spectroscopy and high-performance liquid chromatography (HPLC) solvents were purchased from commercial suppliers and used without prior purification. All experiments involving microbial cultures were performed as independent biological replicates. The following abbreviations are used throughout: 3-hydroxybutyrate (3-HB), 3-nitrophenylhydrazine (NPH), 1-ethyl-3-(3-dimethylaminopropyl) carbodiimide (EDC), trifluoroacetic acid (TFA), sodium hydroxide (NaOH) and isopropyl β-D-1-thiogalactopyranoside (IPTG), polymerase chain reaction (PCR).

1.1 Molecular biology techniques

All genes used in this study were ordered as lyophilised double stranded DNA (ThermoFischer™ or Twist Biosciences™). Single strand oligonucleotide primers were synthesised by Integrated DNA technologies.

For PCR, all the reactions were carried in a 50 µL volume using Phusion® high fidelity DNA polymerase (New England Biolabs™), with a 0.1 ng final DNA template concentration. PCR gene products were gel purified using Qiagen™ gel extraction kit, while recombinant plasmid DNA was purified with a Miniprep kit (Qiagen™) from a 5 mL culture of *E. coli* DH5α harbouring the plasmid of interest. Colony PCR was carried out using OneTaq 2x premix (New England Biolabs) 25 µL reaction scale using the protocol provided by the manufacturer.[1] All restriction enzymes were purchased as Fast Digest™ (ThermoFisher) enzymes. Reactions were performed at 37 °C for 1 hour using 1 µg of DNA template using the protocol provided by the manufacturer.[2] DNA ligations were performed using T4 ligase (ThermoFisher) at 22 °C for 90 minutes, with a 3:1 insert:vector ratio. All plasmids assembled in this work were verified by sequenced by Sanger sequencing at Genewiz (Azenta Life Sciences).

PCRs were performed using the following conditions: initial denaturation at 98 °C for 30 seconds, 30 thermocycles (10 seconds denaturation at 98 °C, annealing at 45 – 72 °C for 30 seconds, and extension at 72 °C for 30 seconds) and a final extension at 72 °C for 5 minutes. All the colony PCRs were performed using the following protocol: initial denaturation (95 °C for 30 seconds), 35 thermocycles (30 seconds denaturation at 95 °C, annealing at 45 – 68 °C for 30 seconds and extension at 72 °C for 60 seconds), and a final extension at 72 °C for 5 minutes. For agarose gel electrophoresis, 2% w/v agarose TAE gels were run at 100 V for 45 minutes and visualised using SYBRSafe™ (Invitrogen). For SDS-PAGE, NuPAGE™ 4-12% Bis – Tris polyacrylamide gels (ThermoFisher) were run at 150 V for 1 hour in 1x MES buffer. Gels were stained using InstantBlue® Coomassie Protein Stain (Abcam) following the standard protocol provided by the manufacturer.

Chemically competent *E. coli* BL21 (DE3) and *E. coli* DH5α cells were transformed with the desired plasmid via heat-shock at 42 °C for 55 seconds and recovered in 200 µL of SOC media for 1 hour at 37 °C. Transformants were plated and selected for on LB agar containing appropriate antibiotics and incubating at 37 °C overnight.

1.2 Toxicity assays

*E. coli*_pHBH_Ac was cultured in LB containing kanamycin (50 µg/mL) and ampicillin (100 µg/mL) at 37 °C, 200 rotations per minute (rpm) overnight and 20 µL of the resulting culture used to inoculate 180 µL LB containing kanamycin (50 µg/mL) and ampicillin (100 µg/mL) containing 3-HB or acetone (0-200 mM) in a 96-well plate. The culture was incubated at 30 °C with 300 rpm orbital shaking in a plate reader (SPECTROstar Nano Microplate Reader, BMG Labtech), measuring absorbance at 600 nm every 5 minutes for 60 hours.

1.3 3-HB as a sole carbon source

*E.coli*_EpRSF, *E.coli*_pAC and *E.coli*_pHBDH^{Pf} strains were cultured in LB containing kanamycin (50 µg/mL) at 37 °C, 200 rpm until an OD_{600} of 0.6, and then heterologous protein expression was induced with IPTG (0.1 mM final concentration) for 3 hours. An aliquot of 1 mL of the culture was collected, centrifuged (10 minutes, 15,700 g, 4 °C) and the pellet was washed twice with fresh M9 media without added carbon source. After the washing step, the pellet was resuspended to an OD_{600} and 20 µL of the solution was used to inoculate, in triplicates, 180 µL of fresh M9 media kanamycin (50 µg/ml) supplemented with a carbon source (glucose to 0.5% w/v final concentration and 3-HB to 25 mM final concentration) to a starting OD_{600} of 0.05 in a 96-well plate. A control of culture without carbon source and fresh media was also included. Cultures were incubated at 37 °C with 300 rpm orbital shaking in a plate reader (SPECTROstar Nano Microplate Reader, BMG Labtech) and absorbance measured at 600 nm every 5 minutes for 18 hours.

1.4 Sample analysis via NMR spectroscopy

A 500 µL aliquot of the culture supernatant was mixed with 500 µL of deuterated chloroform ($CDCL_3$), vortexed then clarified by centrifugation (5 minutes, 15,700 g, 22 °C). The organic phase was transferred to a fresh Eppendorf tube and the aqueous phase was extracted again using the same protocol. The combined organic phases were transferred into a clean Norell® Standard Series™ NMR tube and ^{1}H and ^{13}C NMR spectra were obtained using a PRO500 NMR Spectrometer at 298 K. Proton chemical shifts are expressed in parts per million (ppm, δ scale), referenced to residual protium in the NMR solvent[3] ($CDCl_3$, ∂ 7.26 ppm) and analysed using MNova™ software.

1.5 HPLC sample preparation from fermentation samples

The derivatized mixture was analysed by High Performance Liquid Chromatography (HPLC) using an Agilent 1100 Series HPLC instrument and a Zorbax Eclipse Plus C_{18} column (4.6 x 100 mm, 3.5 µm). In a 96-well plate, 190 µL of methanol 22.5% was added along with 2 µL of caffeine solution (10 mM in 0.1% v/v TFA in water, final internal standard concentration of 100 µM). Then, 8 µL of the derivatization mixture was added. The equipment sampler was kept at 4 °C and the column temperature at 55 °C. All analytes were detected at 250 nm, 280 nm and 350 nm, and then quantified by comparison to a caffeine internal standard. For each sample, a 15 µL volume was injected and analysed using the following gradient with a 0.4 mL/min flow rate:

Time (min)	%B
0	5%
0 to 17	5% to 75%
17 to 20	75% to 5%

A calibration curve was obtained (Figure S18) using a starting solution of 1 M 3-HB (in TB media), which was diluted to known concentrations between 0 mM and 100 mM. Samples were derivatized and analysed in triplicates using the protocol described above. Exemplar HPLC chromatograms of derivatized samples are shown in Figure S19.

2 Additional experimental data

2.1 NMR spectra for acetone production from 3-HB

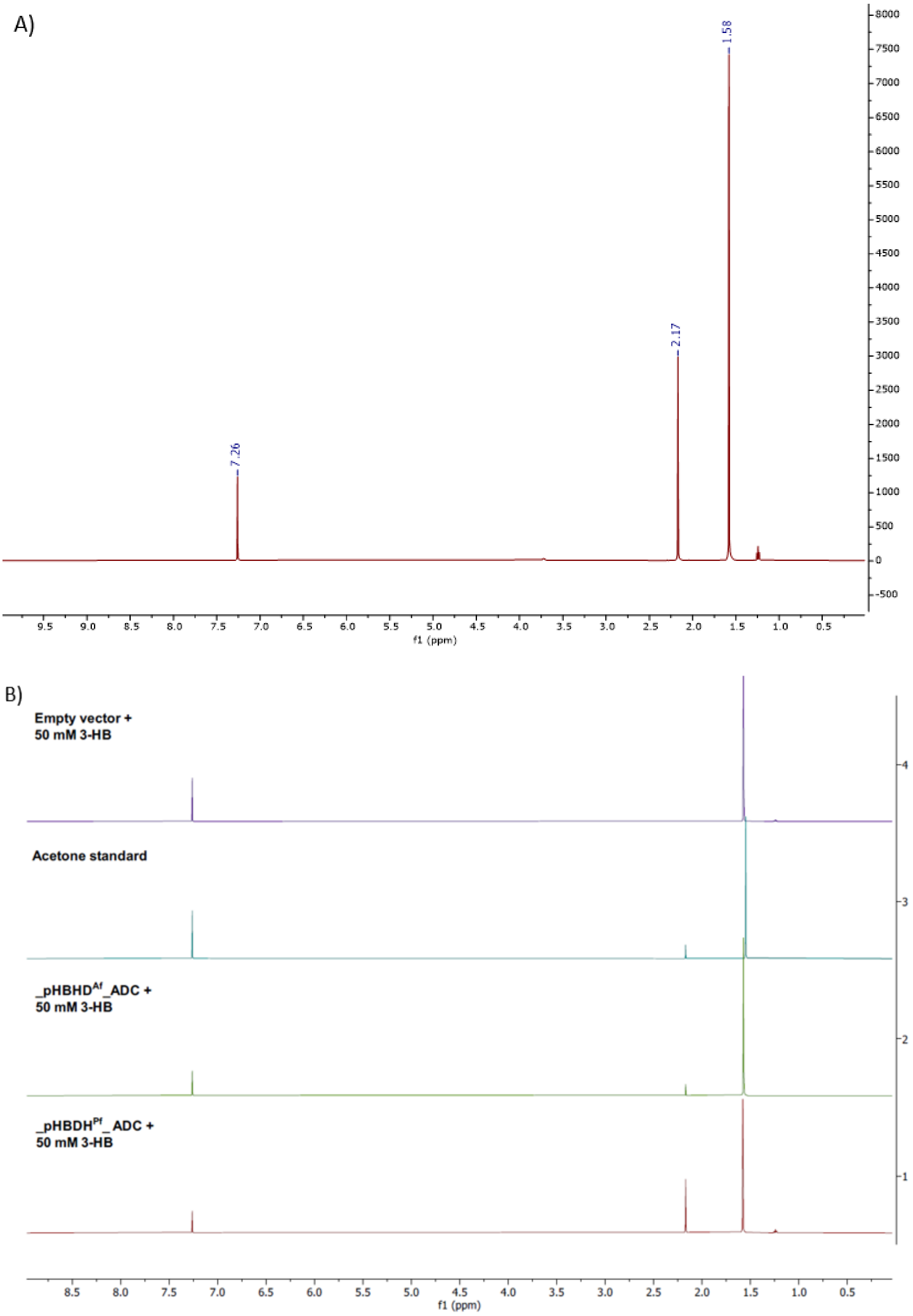

Figure S1: (a) ^{1}H NMR spectrum of whole cell biotransformation from 3-HB to acetone with *E.coli*_pHBHD^{Pf}_ADC after extraction with $CDCl_3$. (b) Alignment of ^{1}H NMR spectra with a commercial standard of acetone and whole cell biotransformation reactions of *E.coli*_pHBHD$^{Af/Pf}$_ADC strains. Peaks: $CDCL_3$: 7.26 ppm, acetone: 2.17 ppm, water: 1.58 ppm.

2.2 Analysis of acetone reduction to isopropanol

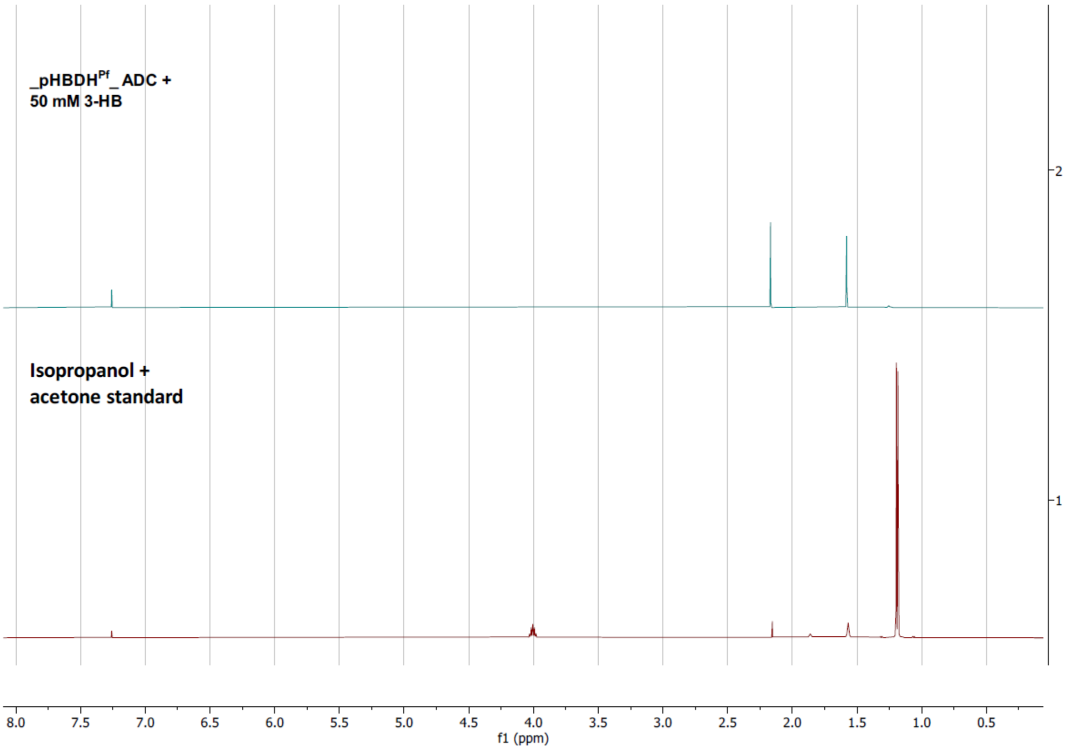

Figure S2: Alignment of ^{1}H NMR spectra with a commercial standard of isopropanol and whole cell biotransformation reaction with *E.coli*_pHBHD^{Pf}_ADC.

2.3 SDS-PAGE analysis of 3-HBDH expression cultures

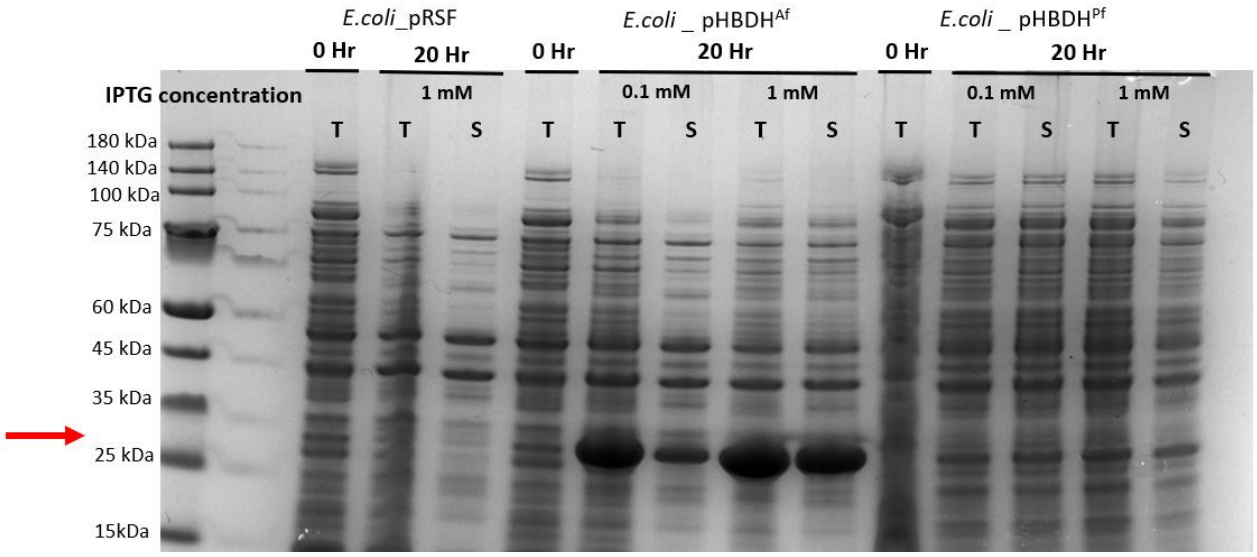

Figure S3: SDS-PAGE analysis for separate expression of 3-HBDH$^{Af/Pf}$ variants and empty vector control (pRSF). T: total protein fraction. S: soluble protein fraction. Theoretical mass of proteins: 3-HBDH^{Af}: 27.1 kDa; 3-HBDH^{Pf}: 26.6 kDa. The red arrow indicates the expected band size.

2.4 SDS-PAGE for PHB depolymerase expression

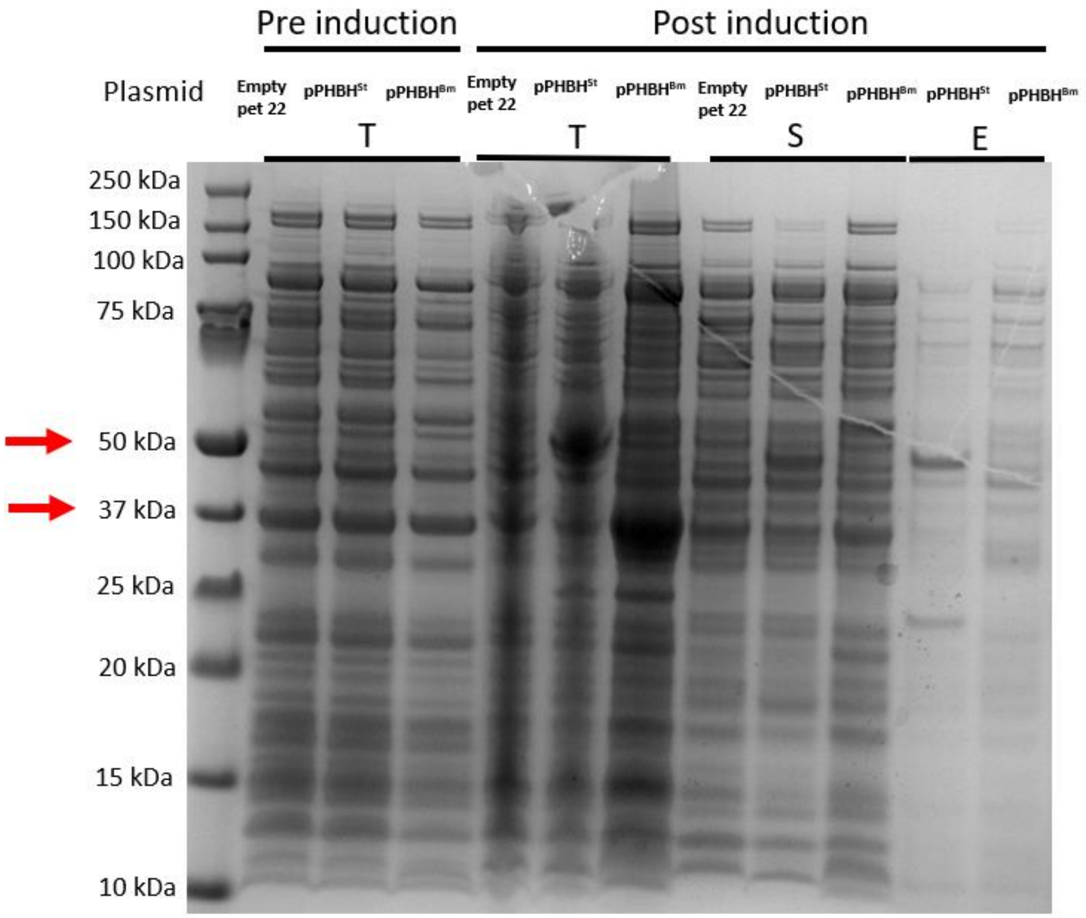

Figure S4: SDS-PAGE of the protein fractions obtained for each culture in LB media 0.1mM IPTG and 0.2% v/v arabinose. E: Extracellular protein. T: Total protein fraction. Theoretical mass of proteins: $PHBH^{St}$: 49.4 kDa, $PHBH^{Bm}$: 38.6 kDa. The red arrow indicates the expected band size.

2.5 PHB degradation assay

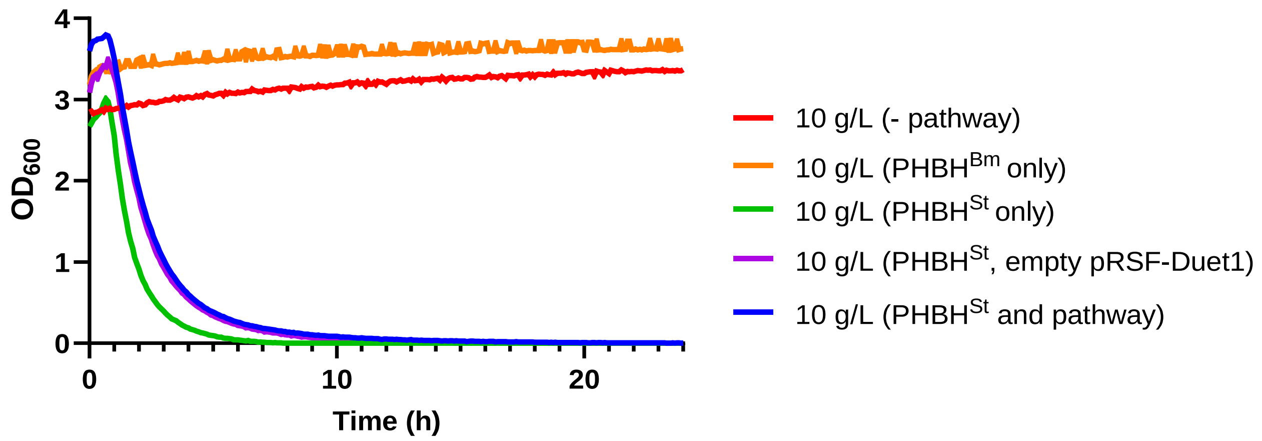

Figure S5: Turbidimetric PHB degradation assay with supernatant of *E. coli* PHB degrading strains. Conditions: 600 µL of filter sterilized supernatant supplement with streptomycin (50 µg/mL) was incubated for 60 hours at 30 °C 300 rpm orbital shaking.

2.6 PHB degradation time

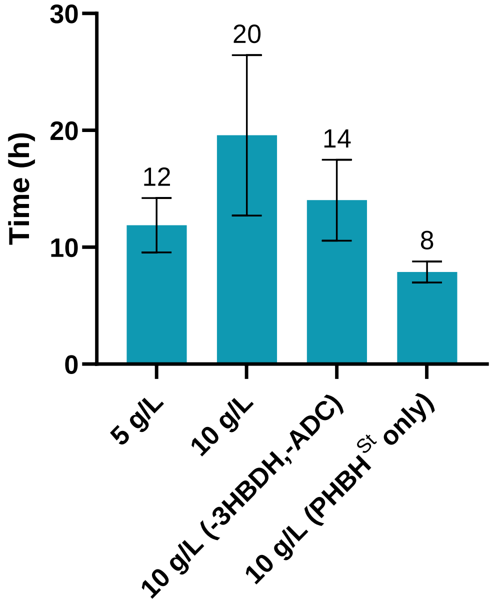

Figure S6: Average time required to achieve complete degradation by PHB degrading *E. coli* strains. Complete degradation was assumed when the OD_{600} = 0 after blank correction. Conditions: 600 µL of filter sterilized supernatant supplement with streptomycin (50 µg/mL) was incubated for 60 hours at 30 °C with 300 rpm orbital shaking.

2.7 Proof of concept of acetone production from PHB

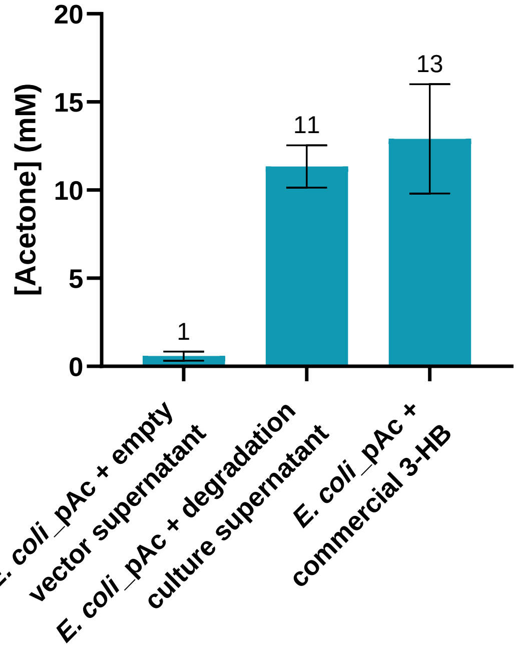

Figure S7: Acetone production from sequential PHB degradation and recycling. Conditions: For all strains, TB media, 37 °C pre-induction temperature, followed by addition of 0.1 mM IPTG and 0.2% v/v arabinose. For degradation strains (*E.coli*_pPHBHSt), 4 hours post induction at 22 °C, 200 rpm and then supernatant incubated at 30 °C, 200 rpm in TB with 3% w/v PHB. *E.coli*_pAC was incubated with filter sterilized supernatant of degradation strain culture (expected 3-HB final concentration of 50 mM) at 22 °C, 200 rpm for 24 hours.

2.8 NMR spectra of acetone production by *E. coli*_pHBDH

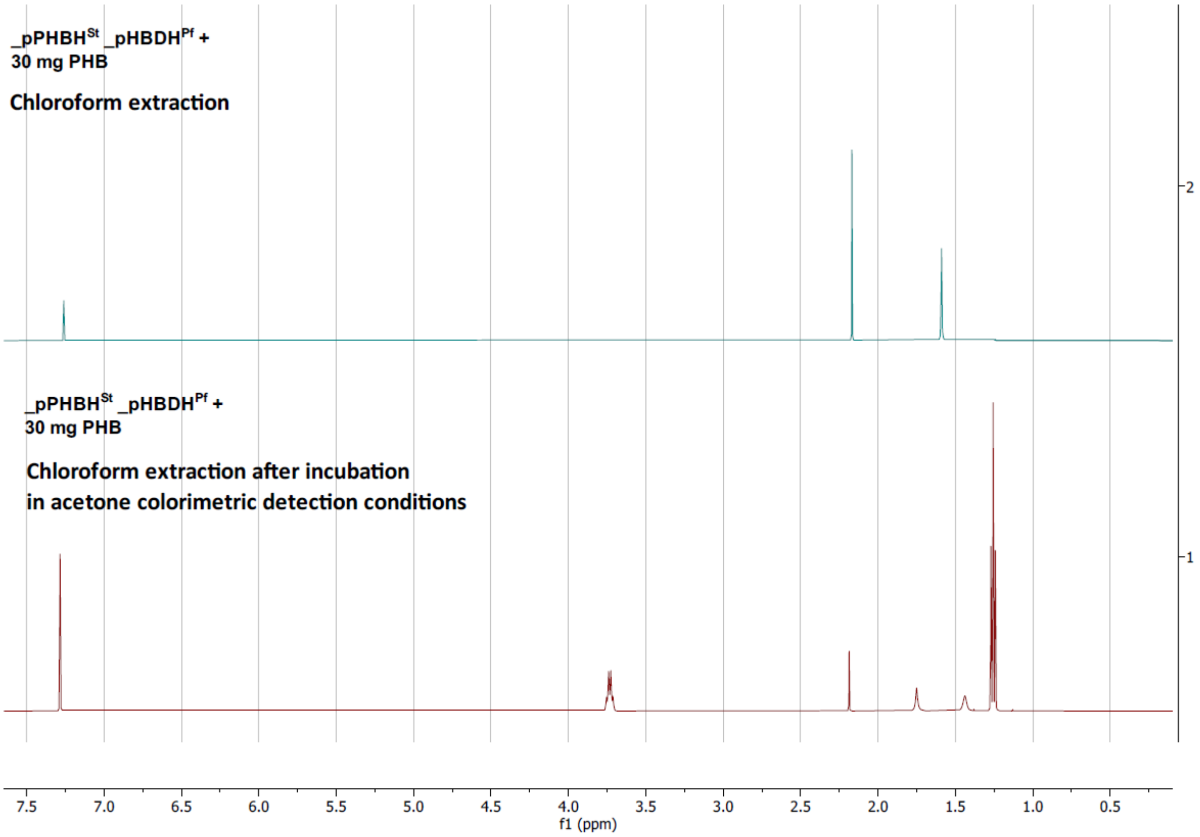

Figure S8: Alignment of ^{1}H NMR spectra of one-pot PHB upcycling reaction with *E.coli*_pPHBHSt strain. The reaction was extracted with d-chloroform before and after acetone colorimetric detection. In both cases, a peak at 2.17 ppm can be observed, indicating the presence of acetone in the cell culture.

2.9 Fermentation media screening for acetone production from 3-HB

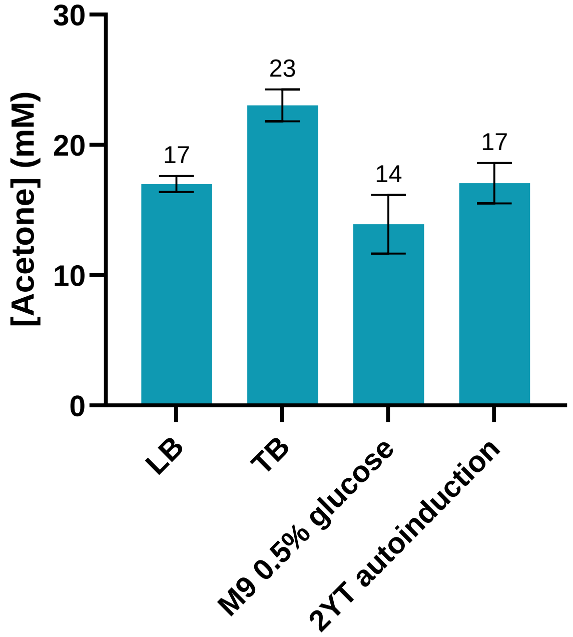

Figure S9: Media screening for acetone production from 3-HB with *E. coli*_pAC strain. Conditions: For all experiments, 37 °C pre-induction temperature, followed by addition of 0.1 mM IPTG and 50 mM final concentration 3-HB, post-induction incubation at 22 °C for 24 hours.

2.10 Additives screening for acetone production from 3-HB

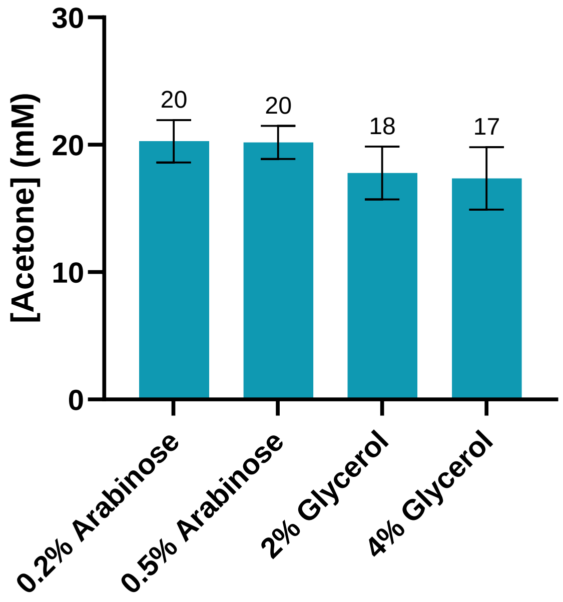

Figure S10: Additives screening for acetone production from 3-HB with *E.coli*_pAC strain. Conditions: LB media, 37 °C pre-induction temperature, followed by addition of 0.1 mM IPTG and 50 mM final concentration 3-HB, post-induction incubation at 22 °C, 200 rpm for 24 hours.

2.11 Effect of IPTG concentration on acetone production from 3-HB

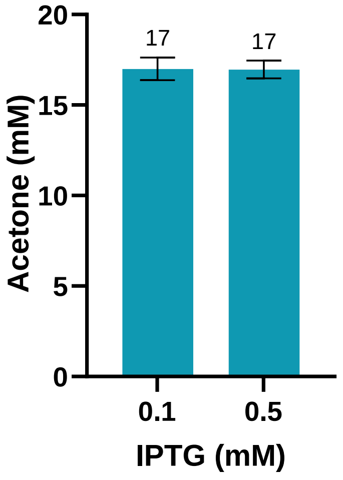

Figure S11: Effect of IPTG concentration in acetone production from 3-HB with *E.coli*_pAC strain. Conditions: LB media, 37 °C pre-induction temperature, followed by addition of 0.1 mM IPTG and 50 mM final concentration 3-HB, post-induction incubation at 22 °C, 200 rpm for 24 hours.

2.12 Effect of reduced pressure on acetone production from PHB

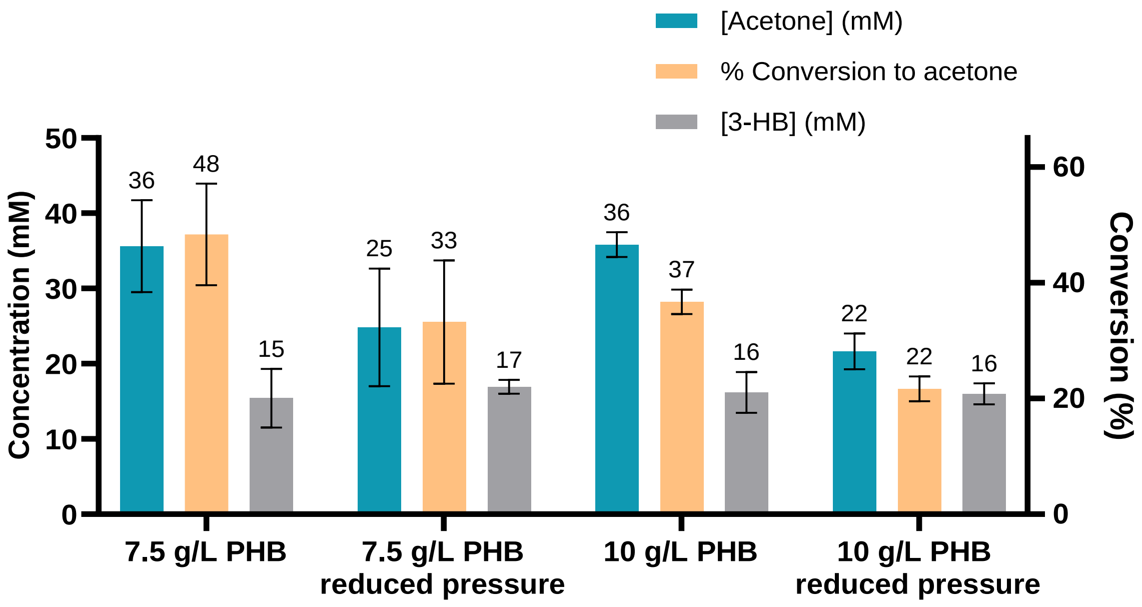

Figure S12: Effect of reduced pressure on acetone production from PHB. Conditions: TB media, 37 °C pre-induction temperature, followed by addition of 0.1 mM IPTG and 2% arabinose. Cultures were performed in 15 mL sterile Hungate tube, post induction incubation at 30 °C, 200 rpm for 24 hours. For reduced pressure conditions, 5 mL of air was removed with a sterile syringe.

2.13 Growth challenge assays with 3-HB and acetone

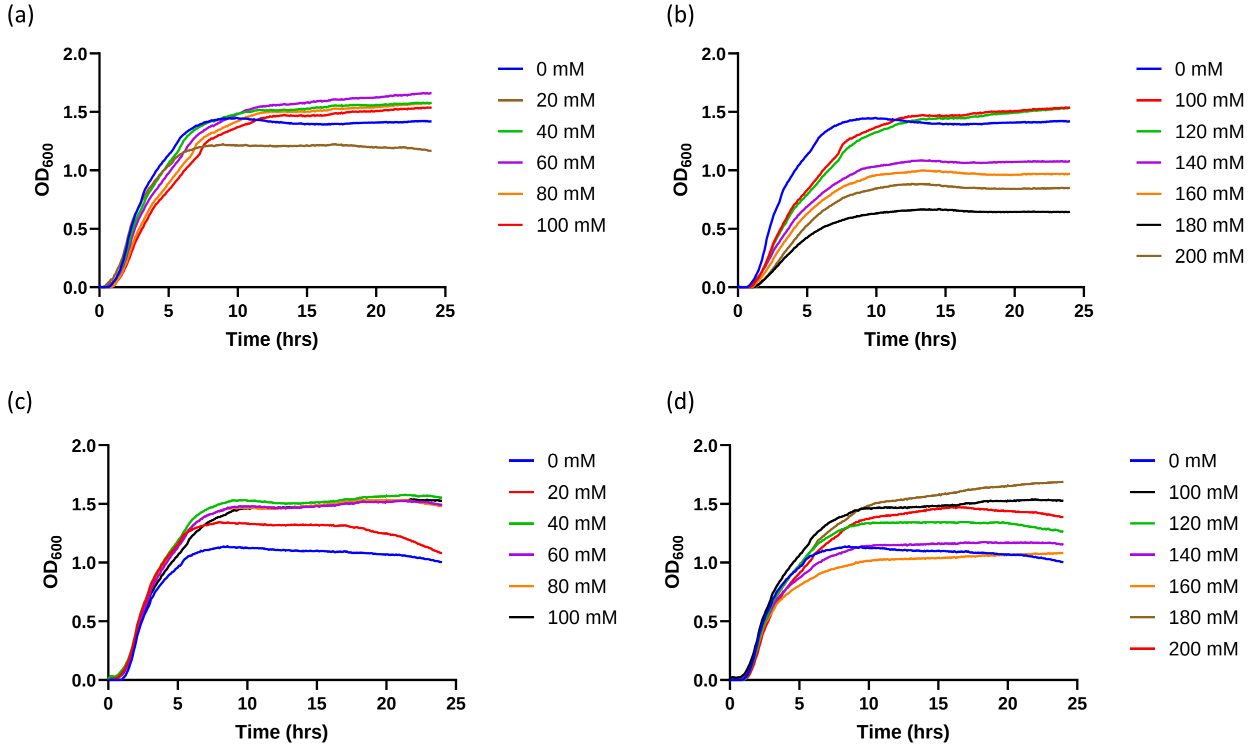

Figure S13: Growth challenge assays with 0-200 mM 3-HB ((a) and (b)) or acetone ((c) and (d)). Conditions: All assays were performed in a 96-well plate, incubated at 37 °C 200 rpm for 60 hours. All conditions were tested as independent biological triplicates. Error bars are not shown to aid data visualization.

2.14 Maximum derivative in toxicity assays with acetone and 3-HB

(a)

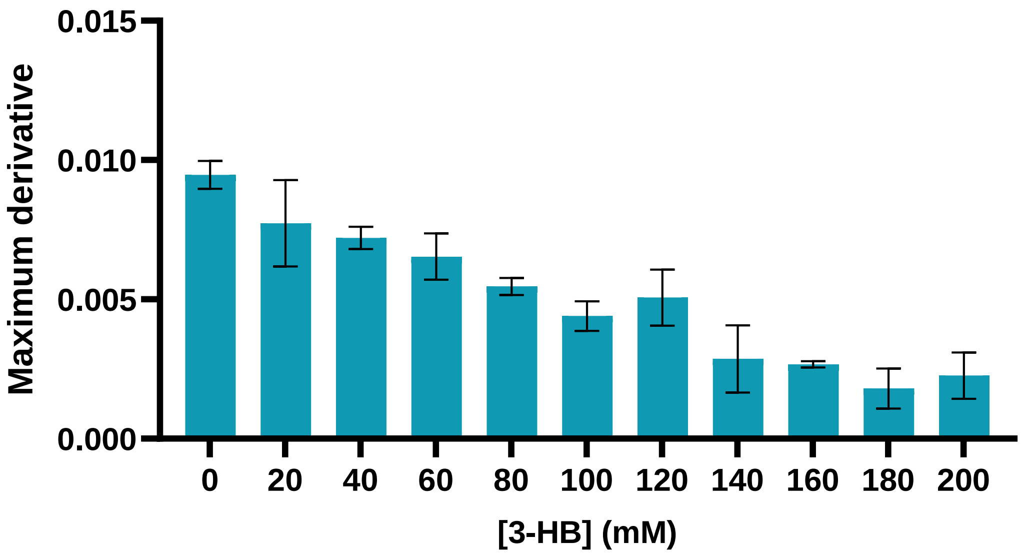

(b)

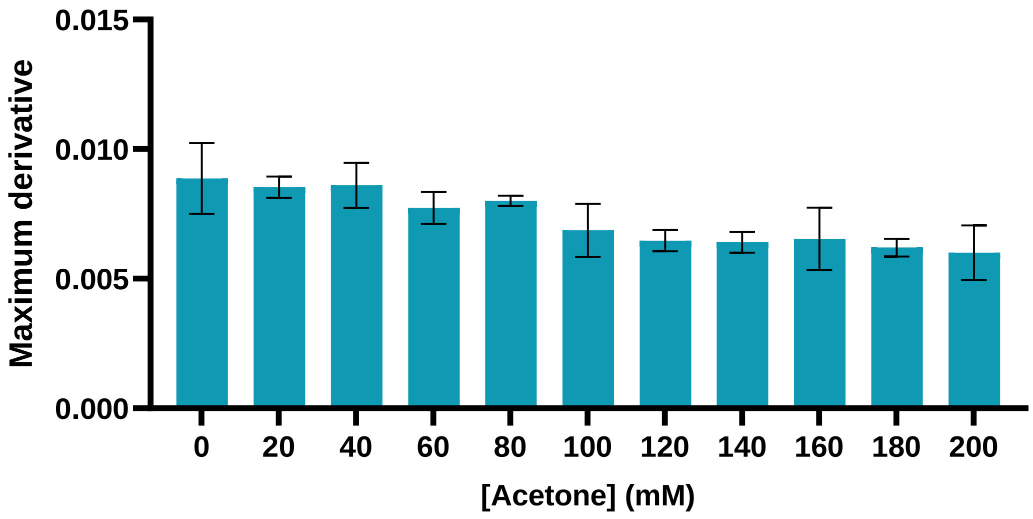

Figure S14: Maximum derivative of growth curves from growth challenge assays using 0-200 mM (a) 3-HB or (b) acetone. All derivatives were calculated in exponential growth phase, using the measurements obtained at 2 hours, which showed the maximum derivative in the sample without the toxic compound (0 mM).

2.15 3-HB as sole carbon source growth curve

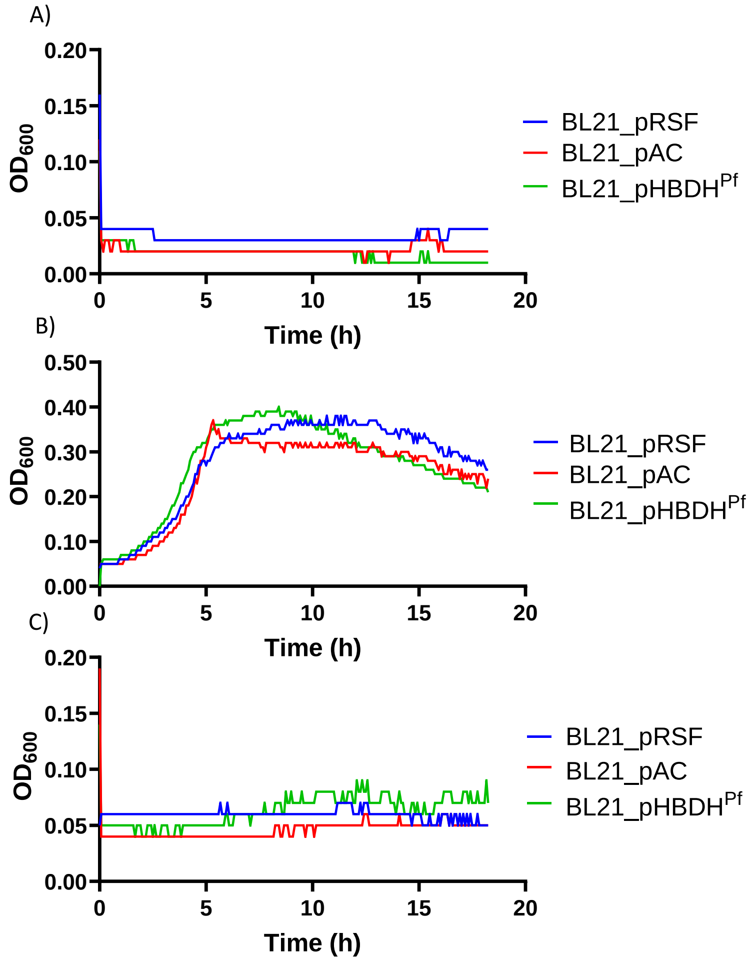

Figure S15: Growth curve of the cells in M9 with different carbon sources. (a) Negative control without carbon source; (b) Positive control with glucose as a carbon source; (c) 25 mM 3-HB. Strain information is detailed in Table S2 and S3.

2.16 NMR spectra of distillate from preparative scale acetone production

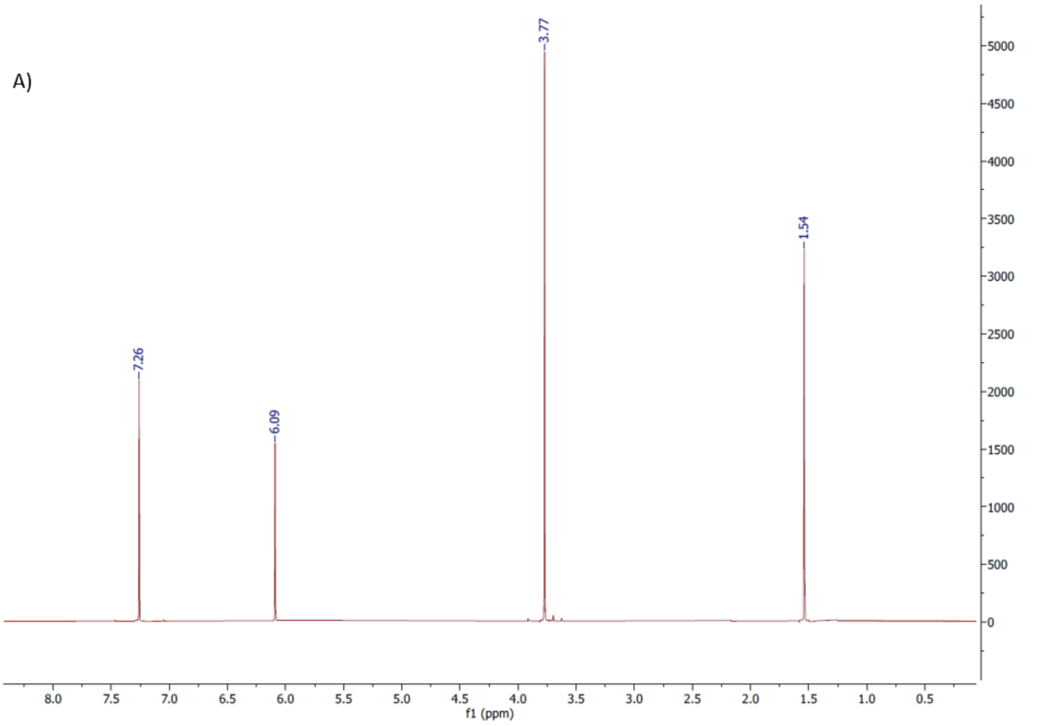

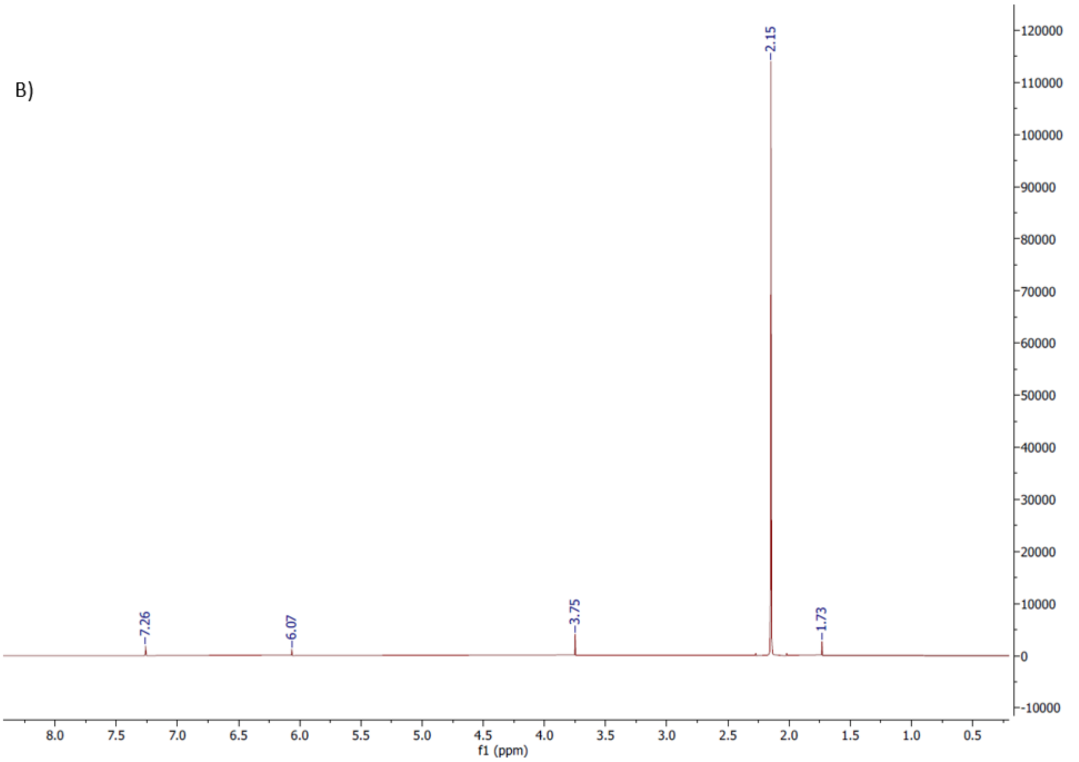

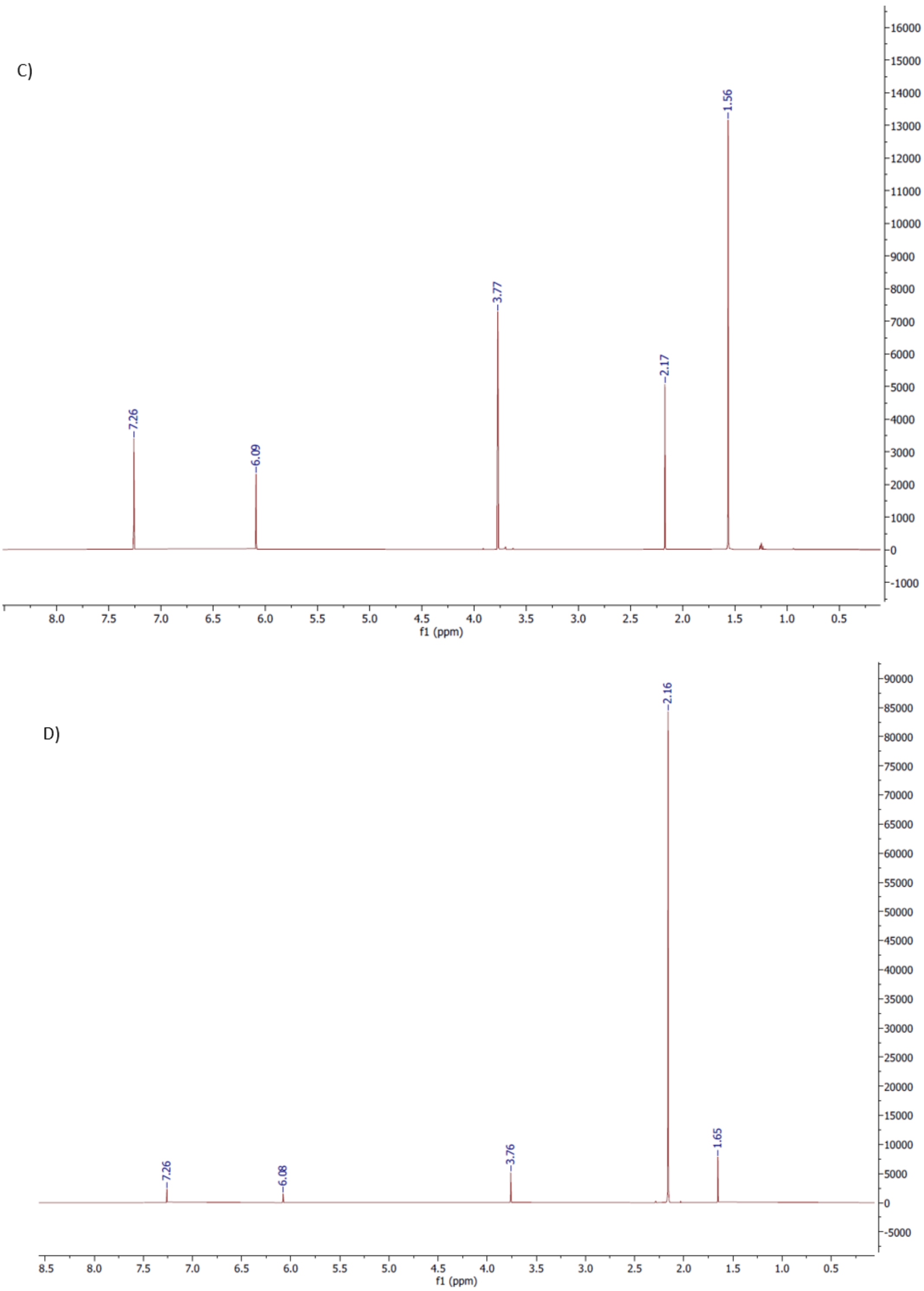

Figure S16: ^{1}H NMR spectra of one-pot PHB upcycling reaction at preparative scale. (a) Chloroform with 5 mM 1,3,5-trimethoxybenzene (TMB); (b) acetone standard; (c) acetone production from commercial PHB; (d) acetone production from post-consumer PHB.

2.17 Calibration curve for colorimetric detection of acetone

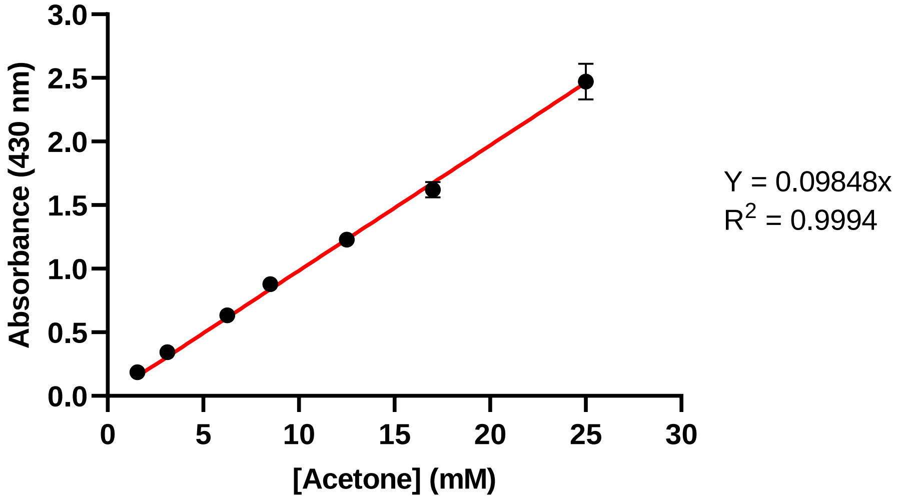

Figure S17: Representative calibration curve for colorimetric acetone detection via derivatisation with vanillin.

2.18 Calibration curve for 3-HB quantification

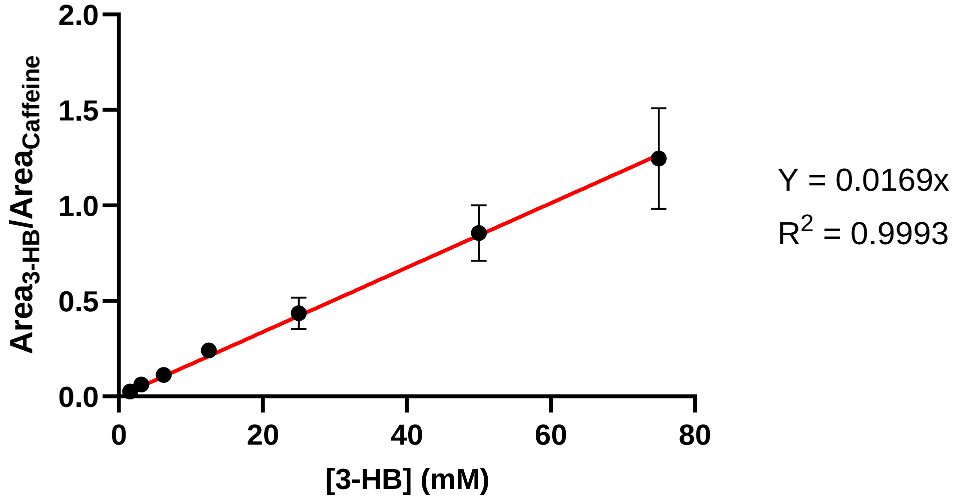

Figure S18: Calibration curve for HPLC quantification of derivatized 3-HB. The x axis shows the starting concentration of 3-HB prior to derivatization, which was performed in the same conditions used for the fermentation samples.

2.19 Exemplar HPLC chromatograms

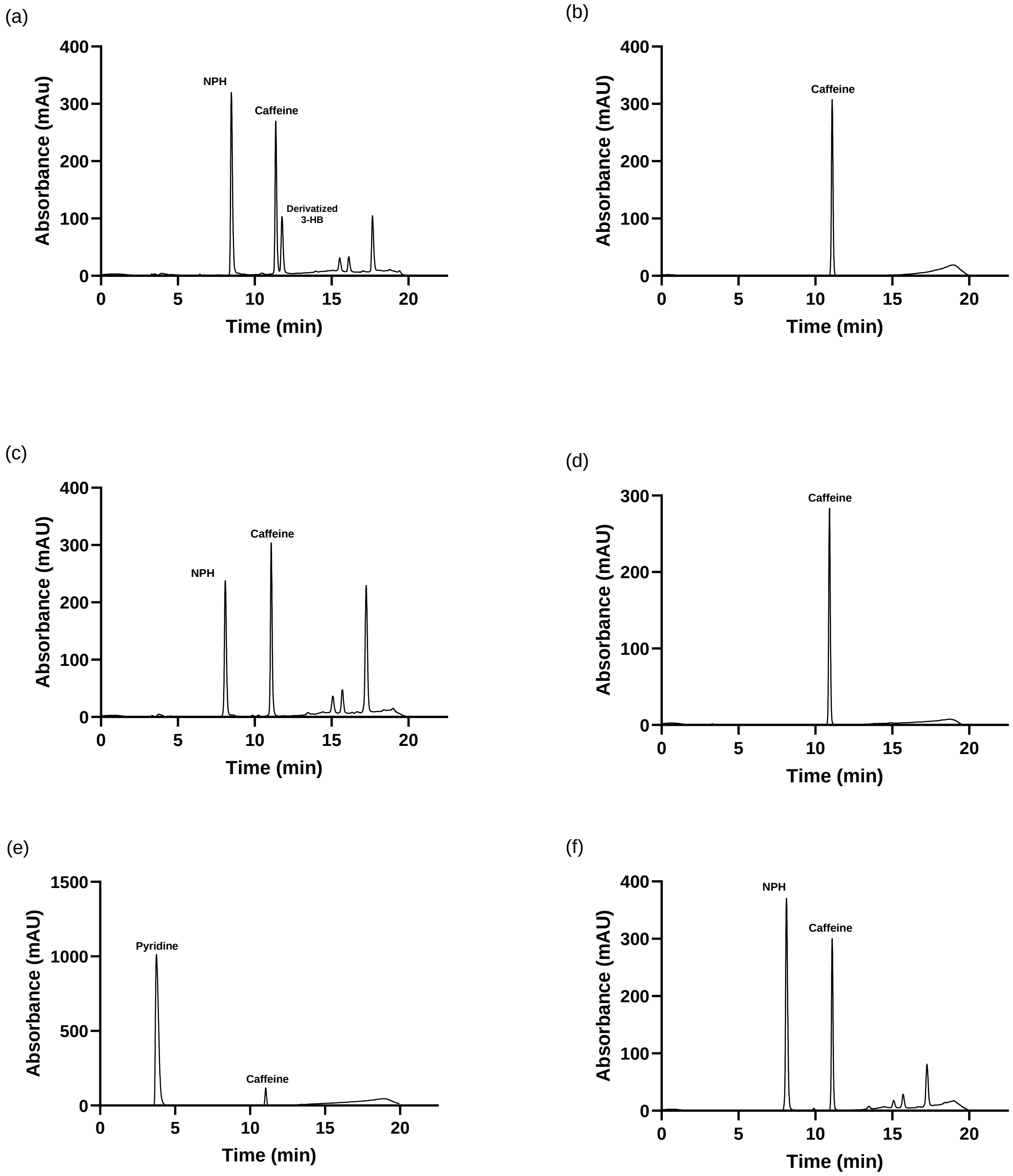

Figure S19: Exemplar HPLC chromatograms of analytes involved in 3-HB derivatisation procedure using caffeine as internal standard and analysed at 280 nm. (a) Derivatization of 250 mM 3-HB; (b) 250 mM 3-HB without derivatization; (c) TB media subjected to derivatisation reaction without 3-HB; (d) Caffeine internal standard; (e), (f), (g) chromatograms of EDC, NPH and pyridine standards, respectively. EDC was not detected in the analysed wavelengths while pyridine was detected at 250 nm.

3 List of sequences, plasmids and strains

3.1 Gene sequences

Table S1. Nucleotide sequences of synthesized gene strings used in this study. Yellow highlight: start codon. Red highlight: stop codon. For PHB hydrolases, the extracellular signal peptide enhancer PelB1 is highlighted in green. Database accession numbers for protein sequences: ADC^{Ca}: Uniprot P23670; 3-HBDH^{Rp}: Genbank AB239334; 3-HBHD^{Af}: NCBI Gene ID: 29369644; 3HBDH^{Pf}: GenBank AB183516. PHBHSt: GenBank MH746934; PHBH^{Bm}: Genbank FJ175152.

Enzyme	Nucleotide sequence (5’ to 3’)
**ADC^{Ca} (804 bp) Codon optimized for *E. coli.***	GGCCAGTGAATTCGAGCTCGGTACGGATCCCATATGCTGAAAGATGAAGTGATCAAGCA<br>GATTAGCACACCGCTGACCAGTCCGGCATTTCCGCGTGGTCCGTATAAATTCCATAATCGC<br>GAATATTTCAACATCGTGTATCGCACCGATATGGATGCACTGCGTAAAGTTGTGCCGGAA<br>CCGCTGGAAATTGATGAACCTCTGGTTCGTTTTGAAATTATGGCAATGCATGATACCAGC<br>GGTCTGGGTTGTTATACCGAAAGCGGTCAGGCAATTCCGGTTAGCTTTAATGGTGTTAAA<br>GGTGATTATCTGCACATGATGTATCTGGATAATGAACCGGCAATTGCAGTTGGTCGTGAA<br>CTGAGCGCATATCCGAAAAAGCTGGGTTATCCGAAACTGTTTGTTGATAGCGATACCCTG<br>GTTGGCACCCTGGATTATGGTAAACTGCGTGTTGCAACCGCCACAATGGGTTATAAACAT<br>AAAGCACTGGATGCCAACGAAGCCAAAGATCAGATTTGTCGTCCGAATTACATGCTGAAA<br>ATCATCCCGAATTATGATGGTAGTCCGCGTATTTGCGAACTGATTAATGCAAAAATTACCG<br>ATGTGACCGTTCATGAAGCATGGACAGGTCCGACACGTCTGCAGCTGTTTGATCATGCAA<br>TGGCACCGCTGAATGATCTGCCGGTTAAAGAAATTGTTAGCAGCAGCCATATTCTGGCCG<br>ATATCATTCTGCCTCGTGCCGAAGTTATTTATGACTACCTGAAATAACTCGAGCTTAAGGA<br>TCCTCTAGAGTCGACCTGCAGG
**3-HBDH^{Rp} (842 bp) Codon optimized for *E. coli.***	GGCCAGTGAATTCGAGCTCGGTACAGATCTCCATGGGTCTGCAGGGTAAAACCGCACTG<br>GTTACCGGTAGCACCTGTGGTATTGGTCTGGGTATTGCACAGGCACTGGCAGCACAGGG<br>TGCAAACATTATTGTTAATGGTTTTCGTCGTGCAGATGGTGCACGTCAGCAGATTGCAGC<br>AGCAGGTCAGGTTATTCGTCTGGGTTATCATGGTGCAGATATGAGCAAAGCAAGCGAAA<br>TTGAAGATATGATGCGTTATGCAGAAGCAGAATTTGCAGCAGATATTCTGGTTAACAATG<br>CCGGTATTCAGCATGTTGCAAGCATTGAAGATTTTCCGCCTGAACGTTGGGATGCAATTAT<br>TGCAATTAATCTGACCAGCGCCTTTCATACCACACGTCTGGCACTGCCTGGTATGCGTCAG<br>AAAAATTGGGGTCGTGTTATTAACATTGCAAGCACACATGGTCTGGTTGCAAGCGCACAG<br>AAAAGCGCCTATGTTGCAGCAAAACATGGTATTGTTGGTCTGACCAAAGTTACAGCACTG<br>GAAACCGCACAGAATCGTGTTACCGCAAATGCAATTTGTCCTGGTTGGGTTCTGACACCG<br>CTGGTTCAGAAACAGGTTCAGGCACGTCCGGCACATGGCATTAGCGTTGAACAGGCAAA<br>ACGTGAACTGGTGATTGAAAAACAGCCGAGCGGTCAGTTTGTTACACCGGATGAACTGG<br>GTGCACTGGCCGTTTTTCTGGCAAGCGAAGCAGGTCGTCAGGTTCGTGGTGCAATTTGGA<br>ATATGGCAGGCGGTTGGTTTGCACAGTAAAAGCTTCGATCGGATCCTCTAGAGTCGACCT<br>GCAGG
**3-HBHD^{Af} (854 bp) No codon optimization.**	GGCCAGTGAATTCGAGCTCGGTACAGATCTCCATGGGTCTGAAAGGTAAAAAAGCGGTC<br>GTAACAGGATCGACCAGTGGTATCGGTCTGGCAATGGCCACCGAGTTGGCCAAAGCTGG<br>TGCAGATGTGGTGATCAATGGTTTTGGTCAGCCTGAAGACATCGAACGCGAGCGCAGCA<br>CGCTGGAGTCCAAGTTTGGTGTCAAAGCCTATTATCTGAATGCAGACCTGAGCGATGCAC<br>AGGCCACGCGCGATTTTATTGCCAAGGCGGCAGAGGCCCTGGGCGGTCTGGATATTCTG<br>GTCAATAATGCCGGCATTCAACACACCGCGCCTATTGAAGAGTTCCCGGTTGATAAATGG<br>AACGCCATCATTGCCCTGAACCTGTCGGCCGTATTCCACGGCACCGCGGCGGCCTTGCCC<br>ATCATGCAAAAGCAGGGCTGGGGACGCATCATCAATATCGCCTCGGCTCACGGTCTGGT<br>GGCCTCGGTAAATAAGTCCGCTTATGTGGCTGCCAAGCACGGTGTAGTGGGGCTGACCA<br>AAGTGACCGCTCTGGAAAATGCCGGCAAGGGGATTACGTGCAACGCAATCTGCCCAGGC<br>TGGGTACGTACTCCCTTGGTGGAAAAACAGATTGAAGCTATCAGCCAGCAAAAAGGGAT<br>TGATATTGAAGCCGCCGCTCGCGAGCTGCTGGCTGAAAAACAGCCCTCCCTGCAGTTCGT<br>GACTCCCGAGCAATTGGGTGGTGCTGCTGTGTTCCTGTCCTCTGCCGCTGCAGATCAAAT

	GACAGGTACGACGCTAAGCCTGGATGGCGGCTGGACAGCACGCTAAAAGCTTCGATCGG<br>ATCCTCTAGAGTCGACCTGCAGG
**3-HBHD^{Pf} (854 bp)<br>No codon optimization.**	GGCCAGTGAATTCGAGCTCGGTACAGATCTCCATGGGTCTCAAAGGAAAAGTCGCAGTC<br>GTCACCGGTTCCACCAGCGGGATCGGCCTGGGTATCGCCACCGCGCTGGCCGCGCAGGG<br>CGCCGATATCGTCCTGAACGGCTTCGGCGACGCCGCCGAGATCGAAAAGGTGCGCGCCG<br>GCCTGGCCGCCCAGCATGGCGTCAAGGTGCTGTACGACGGCGCCGACCTGTCCAAGGGC<br>GAGGCCGTGCGCGGCCTGGTCGACAACGCGGTGCGCCAGATGGGCCGCATCGACATCCT<br>GGTCAACAACGCCGGCATCCAGCACACCGCGCTGATCGAGGACTTTCCCACCGAAAAATG<br>GGACGCCATCCTGGCGCTGAACCTGTCGGCCGTGTTCCACGGCACCGCCGCCGCGCTGCC<br>GCACATGAAGAAGCAGGGCTTCGGCCGCATCATCAACATCGCCTCGGCGCACGGCCTGG<br>TGGCCTCGGCCAACAAGTCGGCCTACGTCGCCGCCAAGCACGGCGTGGTGGGCTTCACC<br>AAGGTGACCGCGCTGGAAACCGCCGGCCAGGGCATCACCGCCAACGCCATCTGCCCGGG<br>CTGGGTGCGCACTCCGCTGGTCGAAAAGCAGATCTCGGCGCTGGCCGAAAAGAACGGCG<br>TCGACCAGGAAACCGCCGCGCGCGAACTGCTCAGCGAAAAGCAGCCGTCGCTGCAATTC<br>GTCACGCCCGAACAACTGGGCGGCACGGCCGTCTTCCTGGCCTCCGATGCCGCCGCGCAA<br>ATCACCGGCACGACCGTCTCCGTCGATGGCGGCTGGACGGCGCGCTGAAAGCTTCGATC<br>GGATCCTCTAGAGTCGACCTGCAGG
**PHBH^{Bm} (1128 bp)<br>Codon optimized for *E. coli*.**	GGCCAGTGAATTCGAGCTCGGTACGGATCCCATATGGAACGTGCATGTGTTGCCGTTATG<br>AAATATCTGCTGCCGACCGCAGCAGCGGGTCTGCTGCTGCTGGCAGCACAGCCAGCCAT<br>GGCACTGGGTAGCCTGCCGAATGTGATTAATACCAGCACACTGGTTAGCAGCAGCGAAC<br>AGCAAGAAGCAAGCAGCGAATCATTTAAAACCTATACGCACAAAGGCAAAAAGTTTAAA<br>GTGTACCTGCCGAAAAGCTATCACATCCTGAAAAGTTATCCGCTGATGGTTATGCTGCAT<br>GGTTGTACCCAGAATGCAGATGATTTTGCAATTGGCACCCAGATGAATGAACTGGCAGAT<br>GAAAAAGAATTCATCGTTGTTTATCCGCAGCAGAGCAGCAATAGCAATATGAATAAATGC<br>TGGAACTGGTTTGAACCGCTGCATCAGATTCGTGGTTTTGGTGAACCGGCAATTATTGCA<br>GGTATTGTTAAAAAGGTGCAGAACATGCACTTCATCCAGAAAAACAAAGTGTTTATTGCC<br>GGTCTGAGTGCCGGTGGTGCAATGAGCATTGTTATGGGTGTTACCTATCCGGAACTGTTT<br>GCCGGTATTGGTGTTAGCGCAGGCATTGAATATCAGGCAGCACTGAATGTTTTTAACGCA<br>TATAGCGTGATGGCAAGCAAAGGTCCGAGTCCGGTTAAACAGGGTCGTATTGCATATCA<br>GCAGATGGGTAAACGTGCAAAACCGCTGCCGCTGATTGTTTTTCATGGTAATGCAGATAG<br>CACCGTGAATGTTGCAAATGCAGATCAGGTTGTTCATCAGTGGATTACCACCAATAATCT<br>GGCATATAACGGTCGCATTGATGGTTGGATTCAGGGTTCACCGGTTAAAACCCGTGAAGA<br>GAAAATTCTGTTTGGTCGTGATTATACCACCTATCGCTATGAAAGCAGCAAAGGTGAACT<br>GGCCATTGACAAATACATTATTAACGGTATGGGTCATGCATGGTCAGGTGGTAATAGCGC<br>AGGTAGCTATACCGATCCGAAAGGTCCTAATGCAAGCCGTATTATGTGGGATTTCTTTAT<br>GAGCCAGAAGGTGTAACTCGAGAAGCTTGATCCTCTAGAGTCGACCTGCAGG
**PHBHST (1521 bp)<br>Codon optimized for E. coli.**	GGCCAGTGAATTCGAGCTCGGTACGGATCCCATATGGAACGTGCATGTGTTGCCGTTATG<br>AAATATCTGCTGCCGACCGCAGCAGCGGGTCTGCTGCTGCTGGCAGCACAACCTGCCATG<br>GCCGCAGGTCTGACCCAGGTTACCGGTTTTGGTAGCAATCCGGGTAATCTGACCATGCAT<br>ACCTATGTTCCGGATGGTCTGGCAGCAGGCGCACCGCTGGTTGTTGCACTGCATGGTTGT<br>ACCCAGAGCGCAAGCGATTATTATGCACATAGCGGTTGGCCGAAATTTGCAGATGCCTAT<br>GGTTTTGCCCTGGTTTTTCCGCAGACCACCAGTGCAAATAATGCAAATAGCTGCTTTAACT<br>GGTTCGATAGCGGTGATAGCACCCGTGGTCAGGGTGAAGCACTGAGCATTCGTCAGATG<br>GTTGATGCAGCAGTTGCACGTTATGGTAGCGATACCCGTCGTGTGTATATTACCGGTCTG<br>AGTGCCGGTGCAGGTATGACCGCAAATATGTTAGCAGCATATCCGGATGTTTTTGCCGGT<br>GGTAGCATTGATAGCGGTCTGCCTGCATATTGTGCAACCAGCGTTAGCGCAGCATATACC<br>TGTATGTATAGCCCTCCGAATAAAACACCGGCACAGTGGGGTGATCTGGTTCGTAGTGCA<br>GCACCGGTTGGCACCAGCAGCTGGCCTCGTGTTGCAATTTGGCAGGGCACCAGCGATAC<br>CACCGTTGCTCCGGCAAATGCAACCGAACTGCGTGATCAGTGGACCAATGTTTGGGGTAT<br>TGGTCAGACCCCGAGCCGTACCGAAAGCCTGAGTGGTGATACCACACTGAGCCAGTATG<br>ATGATGCAAGCGGTCGTCCGGCAGTTAGCGTTTATAGCATTAGCGGTATGGCACATGGCC

	TGGCAGTTGCCCCTGGTAGCGGTCCGGATCAGTGTGGCACCACCGGTACATATTATCTGG<br>ATACCATTTGTAGCAGCTATCATACCGCACGTTTTTGGGGTTTAGATGGTGGTGATGGTG<br>GCAGCGGTCAGAATCCTGGTACACTGCCAGCACCGGCAGGCCTGACCGTTGGTGGTACA<br>ACCGATAGCACCGTTAGCCTGAGCTGGAATCCGGTTGATGGTGCGAGCAGCTATACCGTT<br>TATCGTGGTGGCACCAAAGTTGGTACGACCACCTCAACCGCATATACCGATACAGGTCTG<br>GCAACCGGCACCGCCTATGCGTATACCGTTGCAGCAGTGGATGCAGCGGGTGCAGTTGG<br>TACAGTGAGCAGCAGCGTTACCGCAACCACCACCGGCTATACCCCGACCTGTTATACCGC<br>AAACAATTATCAGCATACCACAGCAGGTCGTGCATATCAGAGCGGTGGTTATACCTATGC<br>AACCGGTAGTGGTCAGGCAATGGGTCTGTATAATACCTTTACCACACATACCCTGAAACA<br>GACCGCACCGGGTTATTATGTTCTGGCAGATAGTGGTTGTAGCGCCTAACTCGAGAAGCT<br>TGATCCTCTAGAGTCGACCTGCAGG

3.2 Plasmids

Table S2: Plasmids generated in this study. 3-HBDH^{Rp}: 3-hydroxybutyrate dehydrogenase from *Ralstonia pickettii*; 3-HBDH^{Af}: 3-hydroxybutyrate dehydrogenase from *Alcaligenes faecalis*; 3-HBDH^{Pf}: 3-hydroxybutyrate dehydrogenase from *Pseudomonas fragi*; ADC^{Ca}: Acetoacetate decarboxylase from *Clostridium acetobutylicum*; PHBH^{Bm}: PHB depolymerase from *Bacillus megaterium*; PHBHSt: PHB depolymerase from *Streptomyces sp. SFB5A*.

Plasmid	Gene	Backbone
**pHBDH^{Rp}**	3-HBDH^{Rp}	pRSF-Duet1
**pHBDH^{Af}**	3-HBDH^{Af}	
**pHBDH^{Pf}**	3-HBDH^{Pf}	
**pADC^{Ca}**	ADC^{Ca}	
**pAc**	3-HBDH^{Pf} and ADC^{Ca}	
**pPHBH$^{St/Bm}$**	PHBH$^{St/Bm}$	pET22b

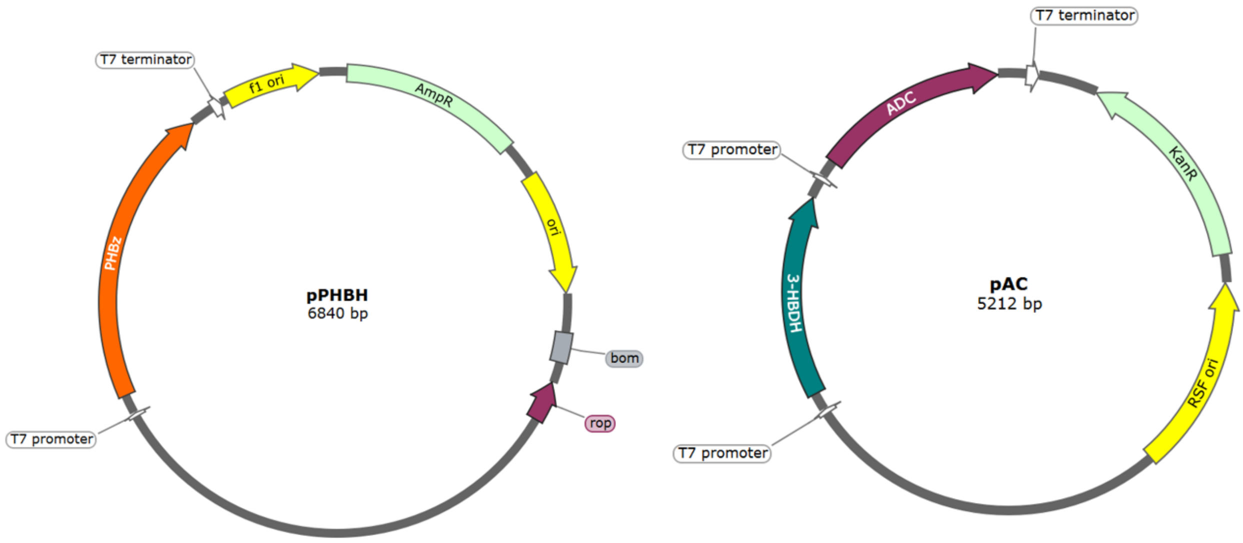

Figure S20: Plasmid maps of pPHBH and pAC.

3.3 Strains

Table S3: Strains used in this work and their abbreviated name.

Strain name	Genotype	Antibiotic resistance
***E. coli*_ pRSF**	BL21 (DE3) Empty pRSF-Duet1	Kanamycin
***E. coli* _ pHBDH^{Rp}**	*E. coli* BL21 (DE3), pHBDH^{Rp}	
***E. coli* _ pHBDH^{Af}**	*E. coli* BL21 (DE3), pHBDH^{Af}	
***E. coli* _ pHBDH^{Pf}**	*E. coli* BL21 (DE3), pHBDH^{Pf}	
***E. coli* _ pADC^{Ca}**	*E. coli* BL21 (DE3), pADC^{Ca}	
***E. coli* _ pAc**	*E. coli* BL21 (DE3), pAc	
***E. coli*_pET22b**	*E. coli* BL21 (DE3) Empty pET22b	Ampicillin
***E. coli*_pPHBHSt**	*E. coli* BL21 (DE3), pPHBHSt	
***E. coli*_pPHBH^{Bm}**	*E. coli* BL21 (DE3), pPHBH^{Bm}	
***E. coli*_pET_pRSF**	*E. coli* BL21 (DE3) Empty pRSF-Duet1, Empty pET22b	Kanamycin and ampicillin
***E. coli*_pPHBH_pAc**	*E. coli* BL21 (DE3), pPHBHSt , pAc	
***E. coli*_x_pAc**	*E. coli* BL21 (DE3) pADC, Empty pET22b	
***E. coli*_pPHBH_x**	*E. coli* BL21 (DE3), Empty pRSF-Duet1 , pAc	

4 References

(1) *PCR Protocol for Taq DNA Polymerase | NEB*. https://uk.neb.com/protocols/0001/01/01/taq-dna-polymerase-with-standard-taq-buffer-m0273 (accessed 2023-08-01).
(2) *T4 DNA Ligase (5 U/µL)*. https://www.thermofisher.com/order/catalog/product/EL0011 (accessed 2023-08-01).
(3) Gottlieb, H. E.; Kotlyar, V.; Nudelman, A. NMR Chemical Shifts of Common Laboratory Solvents as Trace Impurities. *J. Org. Chem.* **1997**, *62* (21), 7512–7515. https://doi.org/10.1021/jo971176v.
